# Structure–Activity Relationship of Natural Dihydrochalcones and Chalcones, and Their Respective Oxyalkylated Derivatives as Anti-*Saprolegnia* Agents

**DOI:** 10.3390/plants13141976

**Published:** 2024-07-19

**Authors:** Alejandro Madrid, Evelyn Muñoz, Valentina Silva, Manuel Martínez, Susana Flores, Francisca Valdés, David Cabezas-González, Iván Montenegro

**Affiliations:** 1 Laboratorio de Productos Naturales y Síntesis Orgánica (LPNSO), Departamento de Ciencias y Geografía, Facultad de Ciencias Naturales y Exactas, Universidad de Playa Ancha, Avda. Leopoldo Carvallo 270, Playa Ancha, Valparaíso 2340000, Chile; evdmunoz@gmail.com (E.M.); silvapedrerosv@gmail.com (V.S.); manuel.m.lobos@gmail.com (M.M.); s.flores.gonzalez@gmail.com (S.F.); fvaldesnavarro@gmail.com (F.V.); 2 Instituto de Química y Bioquímica, Facultad de Ciencias, Universidad de Valparaíso, Av. Gran Bretaña 1111, Valparaíso 2360102, Chile; david.cabezas@postgrado.uv.cl; 3 Escuela de Obstetricia y Puericultura, Facultad de medicina, Campus de la Salud, Universidad de Valparaíso, Angamos 655, Reñaca, Viña del Mar 2520000, Chile; ivan.montenegro@uv.cl

**Keywords:** oxyalkylchalcones, *Adesmia balsamica*, *Saprolegnia parasitica*, *Saprolegnia australis*, SAR studies

## Abstract

*Saprolegnia* sp. is a pathogenic oomycete responsible for severe economic losses in aquaculture. To date, there is no treatment for its control that is effective and does not pose a threat to the environment and human health. In this research, two dihydrochalcones **1** and **2**, and three chalcones **3**–**5**, isolated from the resinous plant *Adesmia balsamica*, as well as their synthesized oxyalkylated derivatives **6**–**29** already reported and a new synthesized series of oxyalkylchalcones **30**–**35**, were evaluated for their anti-*saprolegnia* activity and structure–activity relationship as potential control and treatment agents for strains of *Saprolegnia parasitica* and *S. australis*. Among the molecules tested, natural 2′,4′-dihydroxychalcone (**3**) and new oxyalkylchalcone **34** were the most potent anti*saprolegnia* agents against both strains, even with better results than the commercial control bronopol. On the other hand, the structure–activity relationship study indicates that the contributions of steric and electrostatic fields are important to enhance the activity of the compounds, thus the presence of bulky substituents favors the activity.

## 1. Introduction

Species belonging to the genus *Saprolegnia* were long considered aquatic fungi; currently, it is known that they belong to the kingdom Chromista, which includes brown algae and diatoms [1]. *Saprolegnia* belongs to the division of Oomicota, which houses mostly saprophytic or parasitoid microorganisms. The species of this division have zoospores with barbed and smooth flagella [2]. The Saprolegniaceae Family has, as general characteristics, a highly branched coenocytic mycelium and a cell wall made up mainly of glucans and cellulose, in which septa appear to separate the reproductive organs. Its habitat is mainly aquatic, although it can also live in humid soils. It is tolerant to large temperature ranges from 3 to 33 °C, and its relative salinity tolerance range is approximately 1.75% NaCl [3].

*Saprolegnia* generally travels in colonies consisting of one or more species. They first form a mass of hyphae that, when it grows sufficiently, can be seen with the naked eye, forming a mycelium. Colonies are generally white, turning gray depending on the presence of bacteria or other microorganisms [4]. They release their zoospores into the aquatic environment, which remain dormant until they find a suitable substrate where they begin the infectious process, causing saprolegniosis in fish, amphibians, and crustaceans, both in wild and cultivated environments, threatening biodiversity and food security around the world [5]. One of the most destructive oomycete pathogens in freshwater ecosystems is *Saprolegnia parasitica*, which mainly affects salmonids, from eggs to adult fish. It is characterized by visible patches of white or gray filamentous mycelium on the skin and fins. In severe cases, hyphae invade epidermal tissues, muscles, and blood vessels, ultimately causing the death of infected fish due to osmoregulatory failure and respiratory failure [6]. On the other hand, *S. australis* is responsible for significant mortality of salmonid eggs and fry in fish farms in distant locations, such as Chile and Spain [7], also affecting native North American crustaceans, such as crayfish (*Orconectes propinquus*) [8]; however, this is not the most frequently isolated species from freshwater samples and fish farms [7], but it is no less aggressive.

*Saprolegnia* infection in fish, species-independent, can cause enormous losses in terms of millions of dollars to the aquaculture industry annually [5]. Currently, there are no effective treatments to control *Saprolegnia*. Malachite green was formerly used, but its use has been discontinued worldwide due to its persistence in fish tissues and the environment [9]; later, its successor, formalin, was also banned [10]. The use of veterinary drugs has also declined for the same reason, as they can cause adverse effects to the environment and human health, as well as bacterial resistance due to excessive use of antibiotics [4]. Due to the harmful effects of chemotherapeutic agents, it is necessary to investigate new treatment alternatives that are effective and safe for the environment and the health of the human population. An alternative to current treatments is the use of medicinal plants, such as *Adesmia balsamica* Bertero ex Colla (Fabaceae), known in the vernacular as “paramela de Puangue” and “jarilla” [11]; it is a native and endemic resinous shrub that grows between Aconcagua and Valparaíso provinces in the Valparaíso Region, Chile [12].

Historically, the relevance of “paramelas” has been due to its various uses in traditional medicine to treat rheumatic pains, hair loss [13], colds, digestive disorders [14], and amenorrhea [15], and as an aphrodisiac [16]. *A. balsamica* has the ability to generate large numbers of resinous exudates. It is known that resinous exudates consist of a variety of non-polar compounds, mostly terpenoids and flavonoids [17]. Flavonoids found in exudates are usually chalcone aglycones, flavanones and flavones, which exhibit increased hydrophobicity due to the presence of methoxylated or dehydroxylated substituents on the A- and B-rings compared to the internal tissue forms [18]. Given the large number of bioactive compounds that resinous exudates possess, such as flavonoids and particularly chalcones, they are presented as an alternative for the control of oomycetes, especially considering their lower production cost, biodegradability and non-toxic behavior for the environment. 

Chalcones are secondary metabolites belonging to the flavonoid family. Found naturally in fruits, spices, teas, and soy-based foods, they have received much attention due to their intriguing and potentially beneficial properties [19]. Both natural and synthetic chalcones show high pharmacological potential since they have various biological activities, such as antibacterial [20], anti-inflammatory [21], analgesic [22], antiviral [23], and immuno-modulatory [24], among others. Oxy-prenylated chalcones are an important subclass of natural chalcones [25]. In recent years, it has been shown that they have relevant biological activities, such as antimicrobial activity, which would be provided by the *O*-alkyl chain that increases the lipophilicity of the compounds, allowing greater interaction with cell membranes [26]. On the other hand, for *O*-substituted chalcones as xanthohumol derivatives, anticancer and antioxidant properties have been described in in vitro models [27].

**Figure 1:**
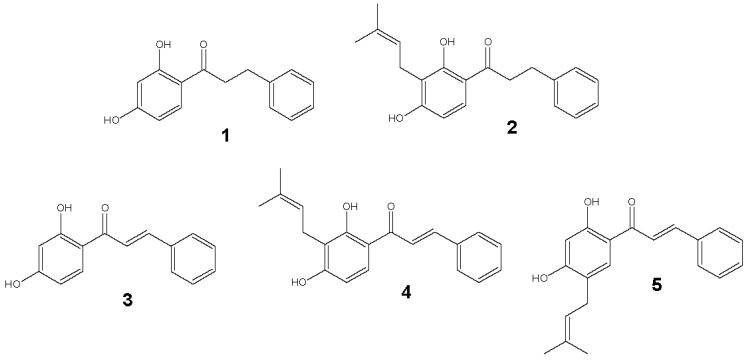
Natural dihydrochalcones **1**, **2** and chalcones **3***–***5** obtained from *Adesmia balsamica*.

Previously, our study group reported the anti-oomycete activity of natural dihydrochalcones: 2′,4′-dihydroxydihydrochalcone (**1**) and dihydroisorcordoin (**2**), as well as their respective chalcones: 2,4-dihydroxychalcone (**3**) and isocordoin (**4**) (see Figure 1). In turn, our group has described for these four natural molecules their respective oxyalkylated derivatives, which have shown strong inhibition of various strains of *Saprolegnia* sp. [25,26,27,28,29,30,31]. In this context, the aim of this study is to report the presence of 2′,4′-dihydroxy-5′ prenylchalcone (**5**) in the resinous exudate of *A. balsamica*, as well as the synthesis of a new series of oxyalkylated derivatives from it, to subsequently perform new anti-oomycete assays against two strains of saprolegniales, *S. parasitica* and *S. australis*, for both the previously described natural and synthetic compounds and chalcone **5** and its synthetic derivatives. In addition, a structure–activity relationship study of all the molecules tested was carried out in order to elucidate the behavior of these types of natural molecule and their oxyalkylated derivatives against *Saprolegnia* strains.

## 2. Results and Discussion

### 2.1. Natural Dihydrochalcones and Chalcones from the Resinous Exudate of Adesmia balsamica

Compounds **1** to **4** were available in the laboratory due to the previous research mentioned above. Compound **5** was isolated in a 3.25% yield from the resinous exudate of *A. balsamica*. Its structure was determined by NMR and the spectroscopic data were compared with the report of this chalcone previously isolated from the aerial parts of *Lonchocarpus cultratus* [32]. On the other hand, these natural dihydrochalcones and chalcones constitute an interesting biosynthetic series, as shown in Figure 1, since they all present a dihydroxylation at the 2′ and 4′ position of the A-ring and the unsubstituted B-ring in their structure.

### 2.2. Synthesis of 4-Oxyalkylchalcones Derivates from Compound ***5***

**Scheme 1:**
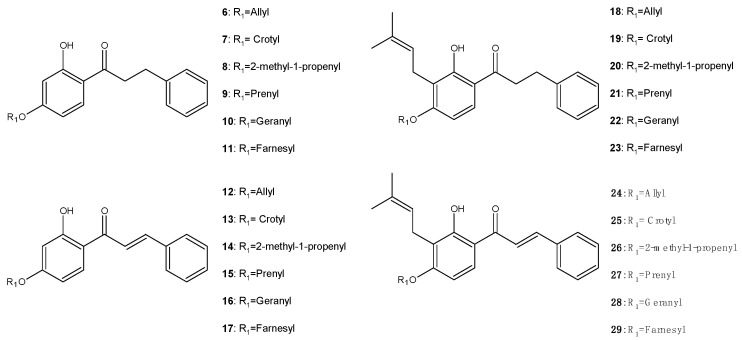
Structure of known dihydrochalcone and chalcone derivatives **6**–**29**.

As described above, all the oxyalkylated derivatives (**6**–**29**, Scheme 1) of the natural molecules **1**–**4**, which are available in our laboratory, were synthesized by a Williamson reaction [25,26,27,28,29,30,31]. 

**Scheme 2:**
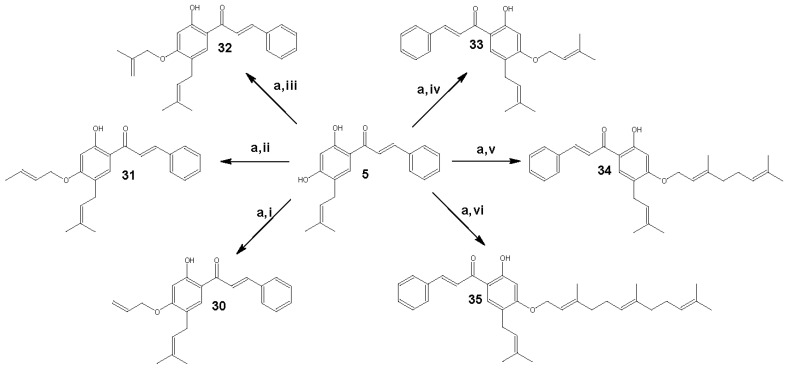
Synthesis of **30**–**35**. Reagents and conditions: (**a**) K_2_CO_3_, dry acetone, reflux at 75 °C for 6 h; (**i**) allyl bromide; (**ii**) crotyl bromide; (**iii**) 2-methyl-1-propenyl bromide; (**iv**) prenyl bromide; (**v**) geranyl bromide; (**vi**) farnesyl bromide.

The new series of 4-oxyalkylchalcones **30**–**35** (Scheme 2), were synthesized through a nucleophilic substitution of the natural chalcone **5** with a series of alkyl bromides by selective alkylation on the hydroxyl located at C-4′. To optimize the preparation of the 4′-mono-*O*-alkylated molecules, different reaction conditions were evaluated, considering the type of solvent, reaction time and temperature, as well as the stoichiometric ratio of the reactants. The conditions that yielded the best products were those with a reaction stoichiometry of 1:1.2:0.5 (natural chalcone/alkyl bromide/potassium carbonate), anhydrous acetone as solvent, and a temperature of 75 °C at reflux for 6 h. The yields obtained for these products ranged from 57.3% for compound **35** to 80% for compound **30**. 

The identification of the new 4-oxyalkylchalcones **30**–**35** depends on the determination of a doublet type signal in the proton spectrum corresponding to the *C*-prenylation on the 5′-carbon of the A-ring of the chalcone, which has a typical chemical shift (δ) for a prenylic chain attached to an aromatic system between 3.0 and 3.05 ppm, which integrates for two protons and presents a coupling constant value (*J*) from 7.0 to 7.2 Hz signal correlated with a signal with δ between 28 and 29 ppm corresponding to the allylic carbon atom of the prenylic chain. The pattern of aromatic substitution of ring A should also be considered, where two singlet signals are observed at 7.85 and 6.40 ppm, corresponding to H-6′ and H-3′, respectively, for chalcones with an unsaturated alpha-beta system, indicating a “*para*” substitution of the aromatic system A. Finally, it is worth mentioning that the *O*-prenylation signal corresponds to a doublet having a chemical shift (δ) typical of an allylic ether-type molecule between 4.50 and 4.60 ppm, which integrates for two protons and has a constant value of coupling (*J*) of 6.2–6.6 Hz signal correlated with a signal with δ between 65 and 72 ppm corresponding to the allylic carbon atom of each alkyl halide.

### 2.3. Anti-Oomycete Activity against S. parasitica and S. australis

To evaluate the anti-oomycete activity of the compounds, the microdilution method described above was used. The commercially used antifungals bronopol, fluconazole and a naturally occurring compound safrole were used as controls.

The results shown in Table 1 demonstrate that compounds **3** and **34** are the most active compounds at 72 h, compared to the controls used. The minimum inhibitory concentration (MIC) value of compound **3** was 52.0 µmol/L against both strains, while the MIC value of compound **34** was 112.5 µmol/L against *S. parasitica* and 56.2 µmol/L against *S. australis*. The minimum oomyceticide concentration (MOC) value for compound **3** was 208.1 µmol/L against *S. parasitica* and 166.4 µmol/L against *S. australis*, while for compound **34** it was 112.5 µmol/L against *S. parasitica* and 56.2 µmol/L against *S. australis*, These results show that compounds **3** and **34** have better activity even than the controls used, such as bronopol, a broad spectrum biocide, which has been used as an effective and economically acceptable alternative in the treatment and control of *Saprolegnia* sp. [31].

In the case of the other compounds evaluated, we could classify them as having moderate activity in relation to compounds **3** and **34**, but higher than the activity of the controls. In this classification are compounds **2**, **24**, **25** and **33**, as can be seen in Table 1. The rest of the 4-oxyalkylated derivatives showed lower anti-*Saprolegnia* activity than the controls used.

Inhibition of mycelial growth was observed by fluorescence microscopy and inverted microscopy. Dehydration and necrosis of the mycelium, as well as the formation of crystals and precipitates, which caused a decrease in the formation of zoosporangium, were considered important damages to the mycelium. The results showed that compound **3** is that which produces a higher percentage of mycelial damage to the *S. parasitica* strains, while compound **1** produces mycelial damage to both *Saprolegnia* strains and reaches 100% damage to *S. australis*. 

A similar effect, although on a smaller scale, was observed for compounds **2**, **8**, **11** and **34**, with percentages of mycelial damage close to or above 50% for both *Saprolegnia* strains. These values are significantly higher than the control used, as shown in Table 2.

Compounds **4**, **5**, **7**, **16**, **19**, **20**, **21**, **26**, **28**, **30**, **31** and **32** showed no activity against any of the strains.

Table 3 shows the cell membrane damage caused by the compounds. This type of test is based on the direct effect of the compounds on sterol formation in fungal or mycobacterial cells and is positive if there is an increase in absorbance at 260 nm in cells treated with the test compounds. Sodium dodecyl sulfate (SDS) is used to compare the effect observed with this chaotropic agent, which causes 100% cell lysis. This assay is performed in triplicate. In this assay, again, the most active compounds were **3** and **34**, showing significantly better activity than the control. Compounds **1**, **2** and **33** also showed moderate activity on both strains, as shown.

### 2.4. 3D-QSAR

#### 2.4.1. Statistical Results of the Models

The results of the internal validation and r^2^_pred_ are presented in Table 4. The *q*^2^ values must be greater than 0.5, and in both cases, this threshold is surpassed. The r^2^_pred_ value must exceed 0.6. In the first model, an r^2^_pred_ of 0.763 was achieved, while the other model resulted in an r^2^_pred_ of 0.831, indicating a high predictive capacity of the model. The predictive values of each model, along with their corresponding residual values, are presented in Table 5. The residual values, obtained by comparing experimental values with predicted values, do not exceed one logarithmic unit. This supports the concept of the model’s predictive capacity. Regarding the contributions of the steric and electrostatic fields, in the first model, the percentage of steric contribution is 58.7%, and the electrostatic contribution is 41.3%. As shown in Table 4, the second model follows the same trend with a larger difference (79.5% and 20.5%, respectively). These results offer insights into the criteria to be employed for the design of new structures.

#### 2.4.2. Contour Maps of CoMFA–SE Models and SAR

**Figure 2:**
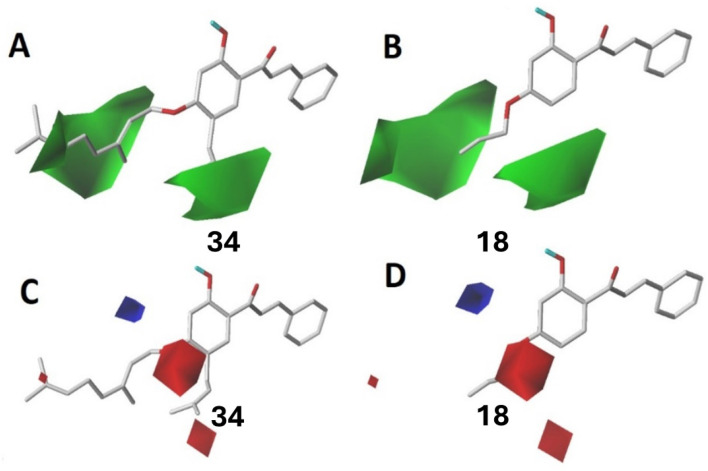
3D–QSAR CoMFA of *Saprolegnia parasitica*. In (**A**,**B**), the steric contour maps are presented, while, in panels (**C**,**D**), the electrostatic maps are displayed.

**Figure 3:**
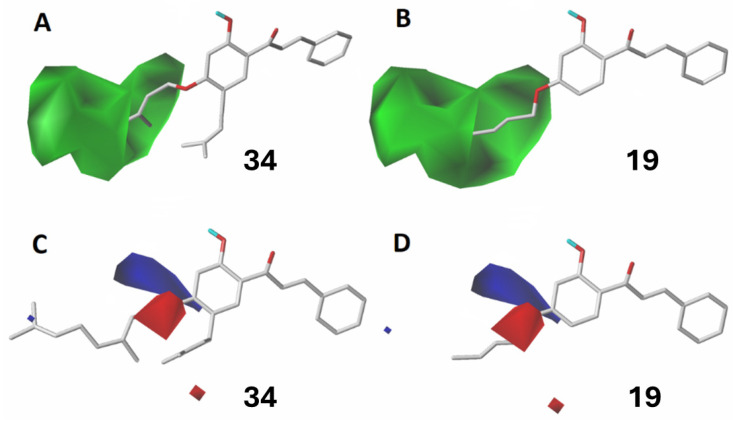
3D–QSAR CoMFA of *Saprolegnia australis*. In (**A**,**B**), the steric contour maps are presented, while, in panels (**C**,**D**), the electrostatic maps are displayed.

In Figure 2 and Figure 3, we present the resulting contour maps of the 3D-QSAR CoMFA steric and electrostatic field models (CoMFA–SE) for *S. parasitica* and *S. australis,* respectively. The steric contour maps for *S. parasitica* are displayed in Figure 2A,B. In panel A, the most active molecule in the model (**34**, pIC_50_ = 3.949; 112.5 µmol/L) is depicted, while the least active is represented in panel B (**18**, pIC_50_ = 3.147; 713.5 µmol/L). In the steric maps, the green poly-hedra indicate that the presence of relatively bulky substituents is favorable for activity. Compound **34** has two bulky groups at the **4′** and **5′** position of ring A, compared to compound **18**, which has one less bulky group than compound **34** at the **4′** position. This difference in activity between these structures may be explained by this observation. As indicated in Table 4, steric contribution holds great relevance for activity. It is suggested to further explore bulky groups in the regions demarcated by the green poly-hedra (**4′** and **5′**). The electrostatic contour map is presented in Figure 2C,D, with 3C depicting the most active molecule in the model and D representing the least active (compounds **34** and **18**, respectively). In this case, the red poly-hedra suggest that the presence of electronegative groups is favorable while the blue poly-hedra indicate that electropositive groups are favorable. In both cases, the presence of the oxygen atom located in the **4′** position of the ring A is favorable. In the isopentyl group region (**5′** of the ring A) of compound **34**, groups with electronegative atoms can be exposed. 

In Figure 3A,B, we have the steric contour map for the *S. australis* model. In panel A, the most active compound in the model (**34**, pIC_50_ = 4.250; 112.5 µmol/L) is depicted, while the least active is represented in panel B (**19**, pIC_50_ = 3.168; 679.5 µmol/L). We observe that the trend seen in the model of *S. parasitica*, as depicted in Figure 3, is repeated. The bulkier group is of greater relevance to the activity. On the other hand, in the electrostatic contour map shown in Figure 3C,D, the same trend is visualized. Figure 3C depicts the most active molecule in the model, while Figure 3D represents the least active compounds (**34** and **19**, respectively). At position **3′**, other groups with electropositive characteristics or low electronegativity can be explored. One option is to add iso-groups or other small hydrocarbon branches. The results discussed in Figure 2 are also applicable to Figure 3.

## 3. Materials and Methods

### 3.1. General

All chemicals and positive controls were obtained from Aldrich (St. Louis, MO, USA) and were used without further purification. Both natural **1**–**4** and synthetic molecules **6**–**29** were available in our laboratory. All reactions were monitored by thin layer chromatography (TLC) on TLC precoated silica gel 60 F254 glass-backed plates (Merck KGaA, Darmstadt, Germany). Flash column chromatography was performed on silica gel (200–300 mesh) (Merck KGaA, Darmstadt, Germany). The ^1^H and ^13^C spectra were recorded in CDCl_3_ solutions and are referenced to the residual peaks of CHCl_3_ at *δ* = 7.26 ppm and *δ* = 77.0 ppm for ^1^H and ^13^C, respectively, on an Avance 400 Digital NMR spectrometer (Bruker, Rheinstetten, Germany) operating at 400.1 MHz for ^1^H and 100.6 MHz for ^13^C. HRMS were recorded in a MAT 95 XL mass spectrometer (Thermo Finnigan, Bremen, Germany).

### 3.2. Isolation of Natural Chalcone from the Resinous Exudate of Adesmia balsamica

The resinous exudate of *A. balsamica* was obtained according to the method previously described by Flores et al. [25], which consists of immersing the fresh branches and leaves of *A. balsamica* (200 g) in dichloromethane for 45 s. The solvent is then removed under reduced pressure, leaving only the extraction products. The resulting resinous exudate (10 g) was fractionated through silica gel chromatographic columns using hexane-ethyl acetate in increasing order of polarity. Compounds **5** were obtained with yields of 3.25%. This procedure was repeated until the critical mass necessary for the development of the subsequent syntheses was obtained.

### 3.3. Synthesis of 4-Oxyalkylchalcones

A solution of natural chalcone **5** (1 mmol), alkyl halides (1.2 mmol), and K_2_CO_3_ (1.5 mmol) in anhydrous acetone (10 mL) was refluxed for 6 h at 75 °C. The end of the reaction is verified by TLC, and then the mixture is poured into ice water (20 mL) and extracted with ethyl acetate (3 × 25 mL). The resulting organic phase was dried over anhydrous potassium sulfate and filtered. The solvent is then evaporated under reduced pressure. All synthesized products were separated and purified by column chromatography (CC) eluting with mixtures of petroleum ether/ethyl acetate of increasing polarity (9.0:1.0 →5.8:4.2). The progress in the separation of the synthetic compounds was analyzed by TLC [33]. The structural determination of the natural chalcone **5** and their derivatives **30**–**35** was confirmed from their spectroscopic properties by NMR. Structural determinations of the previously reported compounds 1–29 can be found in the Supplementary Material.

(2*E*)-1-[4-(allyloxy)-2-hydroxy-5-(prenyl)phenyl]-3-phenylprop-2-en-1-one (**30**): The compound was isolated as a yellow solid in a yield of 80.0% and a melting point of 94–95 °C. ^1^H NMR (400 MHz, CDCl_3_): δ 13.38 (*s*, 1H, **2′**-OH), 7.86 (*s*, 1H, H-**6′**); 7.78 (*d*, *J* = 9.1 Hz, 1H, H-8); 7.62 (*m*, 2H, H-7 and H-4); 7.42 (*m*, 4H, H-2, H-3, H-5 and H-6); 6.47 (*s*, 1H, H-**3′**); 6.07 (*m*, 1H, H-**2″**); 5.44 (*m*, 1H, H-**3″**b); 5.30 (*m*, 1H, H-**3″**a); 5.26 (*m*, 1H, H-**8′**); 4.65 (*d*, *J* = 5.1 Hz, 2H, H-**1″**); 3.03 (*d*, *J* = 7.1 Hz, 1H, H-**7′**); 1.68 (*s*, 3H, H-10′ and H-11′). ^13^C NMR (100 MHz, CDCl_3_): δ 191.3 (C-9); 163.2 (C-**2′**); 162.5 (C-**4′**); 144.1 (C-7); 135.1 (C-1); 132.7 (C-**2″**); 131.8 (C-9′); 130.5 (C-4); 129.1 (C-**6′**); 129.0 (C-2 and C-6); 128.5 (C-3 and C-5); 121.9 (C-**8′**); 120.6 (C-8); 118.0 (C-**5′**) 117.6 (C-**3″**); 114.6 (C-1′); 118.4 (C-**3″**); 103.2 (C-**3′**); 70.0 (C-**1″**); 25.8 (C-**7′**); 21.8 (C-10′); 17.9 (C-11′). HRMS: [M + H]^+^ ion m/z 349.1809 (calcd for C_23_H_24_O_3_, 349.1804).

(2*E*)-1-[4-(crotyloxy)-2-hydroxy-5-(prenyl)phenyl]-3-phenylprop-2-en-1-one (**31**): The compound was isolated as a yellow solid in a yield of 77.1% and a melting point of 93–95 °C. ^1^H NMR (400 MHz, CDCl_3_): δ 13.38 (*s*, 1H, **2′**-OH), 7.85 (*s*, 1H, H-**6′**); 7.77 (*d*, *J* = 9.1 Hz, 1H, H-8); 7.63 (*m*, 2H, H-7 and H-4); 7.42 (*m*, 4H, H-2, H-3, H-5 and H-6); 6.40 (*s*, 1H, H-**3′**); 5.85 (*m*, 1H, H-**2″**); 5.72 (*m*, 1H, H-**3″**); 5.25 (*m*, 1H, H-**8′**); 4.57 (*d*, *J* = 5.9 Hz, 2H, H-**1″**); 3.05 (*d*, *J* = 7.0 Hz, 1H, H-**7′**); 1.68 (*s*, 3H, H-10′ and H-11′); 1.58 (*s*, 3H, H-**4″**). ^13^C NMR (100 MHz, CDCl_3_): δ 191.6 (C-9); 163.2 (C-**2′**); 162.6 (C-**4′**); 144.1 (C-7); 134.9 (C-1); 131.7 (C-9′); 130.5 (C-4); 130.3 (C-**6′**); 129.1 (C-**2″**); 129.0 (C-2 and C-6); 128.5 (C-3 and C-5); 125.6 (C-**3″**); 122.1 (C-**8′**); 120.7 (C-**5′**); 117.8 (C-8); 115.2 (C-1′); 103.7 (C-**3′**); 69.5 (C-**1″**); 25.8 (C-**7′**); 21.8 (C-10′); 17.9 (C-11′); 17.8 (C-**4″**). HRMS: [M + H]^+^ ion m/z 363.1962 (calcd for C_24_H_26_O_3_, 363.1960). 

(2*E*)-1-[4-(2-methylpropenyloxy)-2-hydroxy-5-(prenyl)phenyl]-3-phenylprop-2-en-1-one (**32**): The compound was isolated as a yellow solid in a yield of 76.2% and a melting point of 97–98 °C. ^1^H NMR (400 MHz, CDCl_3_): δ 13.34 (*s*, 1H, **2′**-OH), 7.86 (*s*, 1H, H-**6′**); 7.77 (*d*, *J* = 9.0 Hz, 1H, H-8); 7.63 (m, 2H, H-7 and H-4); 7.42 (*m*, 4H, H-2, H-3, H-5 and H-6); 6.37 (*s*, 1H, H-**3′**); 5.26 (*m*, 1H, H-**2″**); 5.11 (*s*, 1H, H-3b**″**); 5.01 (*s*, 1H, H-3a**″**); 4.55 (*s*, 2H, H-**1″**); 3.03 (*d*, *J* = 7.1 Hz, 1H, H-**7′**); 1.85 (*s*, 3H, H-**4″**); 1.80 (*s*, 3H, H-10′); 1.68 (*s*, 3H, H-11′). ^13^C NMR (100 MHz, CDCl_3_): δ 191.6 (C-9); 163.2 (C-**2′**); 162.8 (C-**4′**); 143.9 (C-7); 141.3 (C-2”); 134.9 (C-1); 131.3 (C-9′); 130.5 (C-4); 129.1 (C-**6′**); 129.0 (C-2 and C-6); 128.5 (C-3 and C-5); 122.0 (C-**8′**); 120.6 (C-**5′**); 117.8 (C-8) 117.8 (C-**3′**); 114.6 (C-1); 113.0 (C-**3″**); 103.2 (C-**3′**); 71.3 (C-**1″**); 25.8 (C-**7′**); 21.8 (C-10′); 19.3 (C-**4″**); 17.8 (C-11′). HRMS: [M + H]^+^ ion m/z 363.1969 (calcd for C_24_H_26_O_3_, 363.1960).

(2*E*)-1-[4-(prenyloxy)-2-hydroxy-5-(prenyl)phenyl]-3-phenylprop-2-en-1-one (**33**): The compound was isolated as a yellow solid in a yield of 79.0 % and a melting point of 102–104 °C. ^1^H NMR (400 MHz, CDCl_3_): δ 13.33 (*s*, 1H, **2′**-OH), 7.85 (*s*, 1H, H-**6′**); 7.77 (*d*, *J* = 9.1 Hz, 1H, H-8); 7.63 (*m*, 2H, H-7 and H-4); 7.42 (*m*, 4H, H-2, H-3, H-5 and H-6); 6.41 (*s*, 1H, H-**3′**); 5.48 (*m*, 1H, H-**2″**); 5.25 (*m*, 1H, H-**8′**); 4.62 (*d*, *J* = 6.6 Hz, 2H, H-**1″**); 3.04 (*d*, *J* = 7.1 Hz, 1H, H-**7′**); 1.80 (*s*, 3H, H-**5″**); 1.78 (*s*, 3H, H-**4″** and H-10′); 1.76 (*s*, 3H, H-11′). ^13^C NMR (100 MHz, CDCl_3_): δ 191.6 (C-9); 163.2 (C-**2′**); 162.8 (C-**4′**); 144.0 (C-7); 138.1 (C-**3″**); 134.9 (C-1); 131.7 (C-**6′**); 130.5 (C-9′); 129.1 (C-4); 129.0 (C-2 and C-6); 128.5 (C-3 and C-5); 122.1 (C-**8′**); 120.7 (C-8); 119.5 (C-**2″**) 117.9 (C-**5′**); 114.5 (C-1′); 103.3 (C-**3′**); 65.2 (C-**1″**); 25.8 (C-**7′**); 25.7 (C-**5″**); 21.8 (C-10′); 18.3 (C-**4″**); 17.8 (C-11′). HRMS: [M + H]^+^ ion m/z 377.2124 (calcd for C_25_H_28_O_3_, 377.2117). 

(2*E*)-1-[4-(geranyloxy)-2-hydroxy-5-(prenyl)phenyl]-3-phenylprop-2-en-1-one (**34**): The compound was isolated as a yellow solid in a yield of 59.0% and a melting point of 81–83 °C. ^1^H NMR (400 MHz, CDCl_3_): δ 13.34 (*s*, 1H, **2′**-OH), 7.85 (*s*, 1H, H-**6′**); 7.77 (*d*, *J* = 9.1 Hz, 1H, H-8); 7.63 (*m*, 2H, H-7 and H-4); 7.42 (*m*, 4H, H-2, H-3, H-5 and H-6); 6.41 (*s*, 1H, H-**3′**); 5.48 (*m*, 1H, H-**2″**); 5.25 (*m*, 1H, H-**8′**); 4.62 (*d*, *J* = 6.6 Hz, 2H, H-**1″**); 3.05 (*d*, *J* = 7.1 Hz, 1H, H-**7′**); 2.10 (*m*, 4H, H-**4″** and H-**5″**); 1.79 (*s*, 3H, H-**7″**); 1.75 (*s*, 3H, H-10′); 1.68 (*s*, 3H, H-11′); 1.61 (*s*, 3H, H-10″); 1.56 (*s*, 3H, H-9″). ^13^C NMR (100 MHz, CDCl_3_): δ 191.6 (C-9); 163.1 (C-**2′**); 162.8 (C-**4′**); 143.9 (C-7); 141.2 (C-**3″**); 134.9 (C-1); 131.9 (C-**6′**); 131.7 (C-**7″**); 130.5 (C-9′); 129.1 (C-4); 129.0 (C-2 and C-6); 128.5 (C-3 and C-5); 123.7 (C-**6″**); 122.1 (C-**8′**); 120.7 (C-**5′**); 119.3 (C-**2″**); 117.8 (C-8); 114.4 (C-1′); 103.1 (C-**3′**); 65.1 (C-**1″**); 39.3 (C-**4″**); 26.3 (C-**7**′); 25.8 (C-**5″**); 25.6 (C-10′); 21.8 (C-10″); 17.8 (C-11′); 17.7 (C-9″); 16.7 (C-**8**″). HRMS: [M + H]^+^ ion m/z 445.2753 (calcd for C_30_H_36_O_3_, 445.2743).

(2*E*)-1-[4-(farnesyloxy)-2-hydroxy-5-(prenyl)phenyl]-3-phenylprop-2-en-1-one (**35**): The compound was isolated as a yellow solid in a yield of 57.3% and a melting point of 74–76 °C. ^1^H NMR (400 MHz, CDCl_3_): δ 13.36 (*s*, 1H, **2′**-OH), 7.85 (*s*, 1H, H-**6′**); 7.77 (*d*, *J* = 9.1 Hz, 1H, H-8); 7.63 (*m*, 2H, H-7 and H-4); 7.42 (*m*, 4H, H-2, H-3, H-5 and H-6); 6.41 (*s*, 1H, H-**3′**); 5.48 (*m*, 1H, H-**2″**); 5.25 (*m*, 1H, H-**8′**); 5.08 (*m*, 2H,H-**6″** and H-10″); 4.65 (*d*, *J* = 6.6 Hz, 2H, H-**1″**); 3.03 (*d*, *J* = 7.0 Hz, 1H, H-**7′**); 2.09 (*m*, 4H, H-**4″**, H-**5″**, H-**8″** and H-9″); 1.79 (*s*, 3H, H-1**2″**); 1.75 (*s*, 3H, H-10′); 1.67 (*s*, 6H, H-11′ and H-1**5″**); 1.62 (*s*, 6H, H-1**3″** and H-1**4″**); ^13^C NMR (100 MHz, CDCl_3_): δ 192.1 (C-9); 163.2 (C-**2′**); 162.9 (C-**4′**); 144.0 (C-7); 141.3 (C-**3″**);135.3 (C-**7″**); 134.9 (C-1); 131.5 (C-9′ and C-1**1″**); 131.3 (C-**6′**); 130.5 (C-4); 129.0 (C-2 and C-6); 128.5 (C-3 and C-5); 124.3 (C-**6″**); 123.6 (C-10″); 122.1 (C-**8′**); 120.7 (C-**5′**); 119.3 (C-**2″**) 116.0 (C-8); 114.4 (C-1′); 103.3 (C-**3′**); 65.1 (C-**1″**); 39.6 (C-**4″**); 39.4 (C-**8″**); 26.7 (C-**7′**); 26.2 (C-9″); 25.8 (C-**5″**); 25.7 (C-1**2″**); 21.9 (C-10′); 17.9 (C-1**5″**); 17.7 (C-11′); 16.7 (C-1**3″**); 16.0 (C-1**4″**). HRMS: [M + H]^+^ ion m/z 513.3376 (calcd for C_35_H_44_O_3_, 513.3369).

### 3.4. Determination of Anti-Oomycete Activity against S. parasitica and S. australis

#### 3.4.1. Oomycete Isolate and Culture Conditions

The strains used in the study were obtained from infected Atlantic salmon captured in Puerto Montt, Chile. They were isolated using previously reported methods [34]. The strains used were ATCC 42030 *S. parasitica* and 38487 *S. australis*. Hyphal growth cultures were performed in petri dishes in yeast dextrose (DY) agar medium and a mixture of antibiotics (oxytetracycline 80%, flumequine 100% and florfenicol 80%) to eliminate the accompanying bacterial biota. The cultures were purified by successive transfers (at least 5 times) using pieces of agar (10 × 10 mm) obtained from the periphery of the growing colony. Plates were then incubated at 18 °C and sub-cultured every 3 days. A reference population was established on a DY gradient and maintained at 4 °C.

#### 3.4.2. Determination of Minimum Inhibitory Concentration (MIC) and Spore’s Germination Inhibition Test

The anti-oomycete activities of all tested compounds were evaluated by dilution test at final concentrations of 3.125, 6.25, 12.5, 25.0, 75.0, 100.0, 125.0, 150.0 and 200.0 µg/L in Griffin sporulation medium [35]. Bronopol, fluconazole and safrole were used as a positive control, while a 1% EtOH/Tween 20 solution was considered as a negative control.

To perform the spore germination inhibition test, the isolated *Saprolegnia* sp. strains are cultured on potato dextrose agar (PDA) plates for 7 to 14 days. After this period, spores were collected from the sporulated colonies and suspended in sterile distilled water (SDW). The concentration of spores in the suspension is determined with a hemocytometer and adjusted to approximately 1 × 10^4^ CFU/mL. Then, 10 µL of the spore suspension were placed on Petri dishes containing the required concentration of compounds to be evaluated in 10 mL PDA and incubated at 25 °C for 72 h. The minimum oomycete concentration (MOC) was determined as the lowest concentration at which the tested chemical compounds prevented visible growth or germination of spores. 

#### 3.4.3. Mycelial Growth Inhibition Test

The in vitro anti-oomycete activities of the evaluated compounds were performed based on the inhibition rate of mycelia growth [31]. The diameter of mycelial growth was measured after inoculation at 25 °C for 48 h. The growth inhibition rate was calculated as shown in Equation (1):

(1) $$\%IR=100\times \frac{\left(x-y\right)}{\left(x-z\right)}$$

where *IR* is the growth inhibition rate; *x*, mycelial growth in control; and the growth of the mycelium in the sample; and *z*, the average diameter of rape seeds.

#### 3.4.4. Measurement of Cell Membrane Lysis

Cell membrane lysis was evaluated according to Lunde [36], measuring the 260 nm absorbing materials released into the medium once the membrane was broken. Cells were cultured with shaking at 30 °C until early stationary phase, then washed twice and diluted to approximately 5 × 10^7^ CFU/mL with cold MOPS buffer, pH 6.0. Cells were aliquoted into tubes and compounds were added from stock solutions at a 100-fold concentration. Cells will be incubated stationary at 30 °C and centrifuged at 8000× *g* for 5 min in microcentrifuge tubes. Lego supernatants were collected for analysis. The results presented are the means of the values of at least three independent tests.

### 3.5. 3D-QSAR

#### 3.5.1. CoMFA Method

CoMFA studies were performed with Sybyl X software version 1.2 [37] installed in a Windows 10 environment on a PC with an AMD Ryzen 7 7700X 8-Core. To acquire the best conformers for each molecule, every compound was drawn in ChemDraw and subjected to a preliminary geometry optimization using MM2 molecular mechanics as is implemented in ChemBio3D software version 12.0. The mol2 structures were imported to Sybyl and MMFF94 charges were assigned to each atom. The minimized structures were superimposed by the atom-by-atom. In addition, a minimization was carried out based on the Powell method [38]. 

To derive the CoMFA descriptor fields, the aligned training set molecules were placed in a three-dimensional cubic lattice with a grid spacing of 2 Å in the x, y, and z directions such that the entire set was included in it. The CoMFA steric and electrostatic field energies were calculated using a sp^3^ carbon probe atom with a van der Waals radius of 1.52 Å and a charge of +1.0. Cut-off values for both steric and electrostatic fields were set to 30.0 kcal/mol.

#### 3.5.2. Internal Validation and Partial Least Squares (PLS) Analysis

PLS analysis was used to construct a linear correlation between the CoMFA descriptors (independent variables) and the activity values (dependent variables) [39]. To select the best model, the cross-validation analysis was performed using the leave-one-out (LOO) method (and sample distance PLS (SAMPLS)), which generates the square of the cross-validation coefficient (*q*^2^) and the optimum number of components (N). The non-cross validation was performed with a column filter value of 2.0 to speed up the analysis and reduce the noise. The *q*^2^ which is a measure of the internal quality of the models, was obtained according to the following Equation (2):

(2) $$q^{2}=1-\frac{\sum {\left(y_{i}-y_{pred}\right)}^{2}}{\sum {\left(y_{i}-\bar{y}\right)}^{2}}$$

where $y_{i}$, $\bar{y}$, and $y_{pred}$ are observed, mean, and predicted activity in the training set, respectively.

### 3.6. Statistical Analysis

The data was reported as mean of at least two independent assays. In the case of a parametric distribution, a One-way ANOVA test was used. If the distribution was not parametric, a Kruskal-Wallis ANOVA test was used. In both cases, the trust level was 95% using the program STATISTICA 7.0.

## 4. Conclusions

The structure–activity relationship of a series of natural dihydrochalcones and chalcones and their respective oxyalkylated derivatives was studied. In this study, the structural identification of compound **5**, obtained from the resinous exudate of *A. balsamica*, was reported for the first time. Six new derivatives **30**–**35** were synthesized from the natural compound. Compounds **1** to **35** showed good anti-oomycete activity against two strains of *Saprolegnia*, highlighting the natural compound **3** and the new synthetic compound **34**. Among the most active compounds, the SAR study indicates that the activity of compound **34** would be given by the contributions of steric and electrostatic fields, given by the two bulky groups in the 4′ (*O*-geranyl) and 5′ (*C*-prenyl) positions of ring A, unlike compound **3**, which only presents two hydroxyl groups in ring A. In conclusion, this family of compounds stands out as potential anti-*Saprolegnia* agents, which can be used as a real alternative to commercial products.

## Figures and Tables

**Table 1:** Minimum inhibitory concentration (MIC) and minimum oomyceticide concentration (MOC) values of evaluated compounds at 72 h.

Compounds	MIC ^a^ (µmol/L)		MOC ^b^ (µmol/L)		Compounds	MIC ^a^ (µmol/L)		MOC ^b^ (µmol/L)	
	*S. parasitica*	*S. australis*	*S. parasitica*	*S. australis*		*S. parasitica*	*S. australis*	*S. parasitica*	*S. australis*
**1**	515.9	412.8	619.2	515.9	**20**	679.4	594.5	>679.4	679.4
**2**	322.2	322.2	322.2	322.2	**21**	648.6	175	>648.6	648.6
**3**	52.0	52.0	208.1	166.4	**22**	531.2	464.8	531.2	464.8
**4**	>648.6	648.6	>648.6	>648.6	**23**	281.1	224.9	149.9	281.1
**5**	>648.5	648.5	>648.5	>648.5	**24**	286.9	215.2	358.7	430.5
**6**	531.3	442.8	708.4	619.9	**25**	275.9	206.9	344.9	275.9
**7**	>674.8	674.8	>674.8	674.8	**26**	344.9	344.9	344.9	344.9
**8**	421.8	421.8	506.1	421.8	**27**	398.4	332.0	332.0	332.0
**9**	>644.3	644.3	>644.3	644.3	**28**	>449.8	>449.8	>449.8	>449.8
**10**	>528.4	528.4	>528.4	528.4	**29**	292.6	146.3	292.6	195.1
**11**	>447.8	>447.8	>447.8	>447.8	**30**	>573.9	573.9	>573.9	>573.9
**12**	356.7	285.3	427.9	356.7	**31**	>550.3	550.3	>550.3	>550.3
**13**	342.9	342.9	411.5	342.9	**32**	551.8	551.8	>551.8	>551.8
**14**	411.5	411.5	411.5	411.5	**33**	265.6	265.6	125	265.6
**15**	>528.4	264.2	>528.4	330.3	**34**	112.5	56.2	112.5	56.2
**16**	>447.8	>447.8	>447.8	>447.8	**35**	292.6	243.8	292.6	243.8
**17**	242.8	194.3	242.8	194.3	**Bronopol**	>1000	875.0	>1000	1000
**18**	713.5	1624.3	713.5	624.3	**Fluconazole**	>653.0	>653.0	>653.0	>653.0
**19**	>679.5	679.5	>679.5	679.5	**Safrole**	1078.9	924.8	1078.9	924.8

^a,b^ Each value represents the mean of three experiments (*p* < 0.05), performed in quadruplicate.

**Table 2:** Percentage of mycelial growth inhibition (MGI) by evaluated compounds at 48 h.

Compounds	MGI (%)		Compounds	MGI (%)	
	*S. parasitica*	*S. australis*		*S. parasitica*	*S. australis*
**1**	55	100	**19**	0	0
**2**	43	45	**20**	0	0
**3**	100	0	**21**	0	0
**4**	0	0	**22**	36	38
**5**	0	0	**23**	40	13
**6**	13	36	**24**	31	33
**7**	0	0	**25**	25	30
**8**	50	43	**26**	0	0
**9**	0	38	**27**	28	30
**10**	10	13	**28**	0	0
**11**	50	55	**29**	37	39
**12**	15	17	**30**	0	0
**13**	10	12	**31**	0	0
**14**	8	10	**32**	0	0
**15**	12	14	**33**	39	41
**16**	0	0	**34**	55	58
**17**	19	23	**D**	20	23
**18**	12	37	**Bronopol**	0	33

Each value represents the mean of three experiments (*p* < 0.05), performed in quadruplicate.

**Table 3:** Percentage of damage produced by evaluated compounds.

Compounds	Damage (%) ^a^		Compounds	Damage (%) ^a^	
	*S. parasitica*	*S. australis*		*S. parasitica*	*S. australis*
**1**	40	50	**19**	0	0
**2**	43	45	**20**	0	0
**3**	100	25	**21**	10	0
**4**	0	0	**22**	23	27
**5**	0	0	**23**	30	35
**6**	0	0	**24**	31	33
**7**	0	0	**25**	25	30
**8**	30	40	**26**	35	38
**9**	0	0	**27**	28	30
**10**	0	0	**28**	0	0
**11**	0	0	**29**	37	39
**12**	15	17	**30**	0	0
**13**	10	12	**31**	0	0
**14**	8	10	**32**	0	0
**15**	12	14	**33**	40	44
**16**	0	0	**34**	58	60
**17**	19	23	**35**	27	29
**18**	16	0	**Bronopol**	0	33

^a^ Damage produced by evaluated compounds compared to the damage produced by SDS. This was utilized at a final concentration of 2%, which produced 100% of cell lysis. The assay was performed in triplicate. Results presented are the means of values from at least two independent assays.

**Table 4:** Statistical results of the 3D-QSAR CoMFA–steric-electrostatic (CoMFA–SE) analysis.

CoMFA–SE								% Contribution	
	N	*q* ^2^	r^2^_NCV_	SEE	F	SEP	r^2^_pred_	Steric	Electrostatic
***S. parasitica***	1	0.547	0.729	0.08	45718	0.103	0.763	0.587	0.413
***S. australis***	2	0.623	0.917	0.05	88692	0.099	0.831	0.795	0.205

**Table 5:** Predicted pIC_50_ activity values and residuals of the 3D-QSAR CoMFA Models.

	pIC_50_ *S. parasitica*				pIC_50_ *S. australis*		
Compound	Exp	Pred	Residual	Compound	Exp	Pred	Residual
*5	3.1881	3.270	−0.082	*5	3.188	3.191	−0.003
**6**	3.2747	3.159	0.116	**6**	3.354	3.147	0.207
**7**	3.1708	3.187	−0.016	**7**	3.171	3.200	−0.029
*8	3.3749	3.197	0.178	*8	3.375	3.174	0.201
**9**	3.1909	3.185	0.006	**9**	3.191	3.188	0.003
*10	3.2770	3.289	−0.012	*10	3.277	3.312	−0.035
**11**	3.3489	3.353	−0.004	**11**	3.349	3.300	0.049
**18**	3.1466	3.157	−0.010	**18**	3.205	3.172	0.033
**19**	3.1678	3.184	−0.016	**19**	3.168	3.224	−0.056
**20**	3.1678	3.196	−0.028	**20**	3.226	3.208	0.018
**21**	3.1881	3.186	0.002	**21**	3.246	3.223	0.023
**22**	3.2747	3.293	−0.018	**22**	3.333	3.342	−0.009
*23	3.5511	3.344	0.207	*23	3.648	3.321	0.327
**32**	3.2582	3.310	−0.052	**32**	3.258	3.320	−0.062
**33**	3.5758	3.393	0.183	**33**	3.576	3.498	0.078
*34	3.9490	3.491	0.458	*34	4.250	3.637	0.613
**35**	3.5338	3.559	−0.025	**35**	3.613	3.635	−0.022

* Test set.

## Data Availability

All data are available on the manuscript or in the Supplementary Material for the scientific community.

## Supplementary Materials

The following supporting information can be downloaded at https://www.mdpi.com/article/10.3390/plants13141976/s1, S1. Spectroscopic data of natural dihydrochalcones and chalcones **1**–**5**. S2. Spectroscopic data and structures of known dihydrochalcones and chalcones derivatives **6**–**29**. S3. ^1^H, ^13^C NMR of new compounds **30**–**35**.
