# Supplementary material for: Structure–Activity Relationship of Natural Dihydrochalcones and Chalcones, and Their Respective Oxyalkylated Derivatives as Anti-Saprolegnia Agents
Source: Plants (Basel). 2024 Jul 19;13(14):1976. doi: 10.3390/plants13141976 (PMC11281103; doi:10.3390/plants13141976)
Supplement: Supplementary file 1 [file plants-13-01976-s001.zip › plants-3101822-supplementary.pdf]

SUPPORTING INFORMATION

Structure–Activity Relationship of Natural Dihydrochalcones and Chalcones, and Their Respective Oxyalkylated Derivatives as Anti-*Saprolegnia* Agents

Alejandro Madrid [1,*], Evelyn Muñoz [1], Valentina Silva [1], Manuel Martínez [1], Susana Flores [1], Francisca Valdés [1], David Cabezas-González [2] and Iván Montenegro [3]

1 Laboratorio de Productos Naturales y Síntesis Orgánica (LPNSO), Departamento de Ciencias y Geografía, Facultad de Ciencias Naturales y Exactas, Universidad de Playa Ancha, Avda. Leopoldo Carvallo 270, Playa Ancha, Valparaíso 2340000, Chile; evdmunoz@gmail.com (E.M.); silvapedrerosv@gmail.com (V.S.); manuel.m.lobos@gmail.com (M.M.); s.flores.gonzalez@gmail.com (S.F.); fvaldesnavarro@gmail.com (F.V.)

2 Instituto de Química y Bioquímica, Facultad de Ciencias, Universidad de Valparaíso, Av. Gran Bretaña 1111, Valparaíso 2360102, Chile; david.cabezas@postgrado.uv.cl

3 Escuela de Obstetricia y Puericultura, Facultad de medicina, Campus de la Salud, Universidad de Valparaíso, Angamos 655, Reñaca, Viña del Mar 2520000, Chile; ivan.montenegro@uv.cl

* Correspondence: alejandro.madrid@upla.cl; Tel.: +56-032-250-0526

Supplementary materials:

S1 Spectroscopic data of natural dihydrochalcones and chalcones 1-5.

S2. Spectroscopic data and structures of known dihydrochalcones and chalcones derivatives 6-29.

S3. NMR ^{1}H and ^{13}C of new synthetic derivatives 30-35.

S1. Spectroscopic data of natural dihydrochalcones and chalcones 1-5.

(*E*)-1-cyclohexa-1,3-dien-1-yl-3-cyclohexa-1,5-dien-1-ylprop-2-en-1-one (**1**): The compound was isolated as a yellow powder with a yield of 0.8 %. ^{1}H NMR (400 MHz, $CDCl_3$): δ 12.79 (1H, 2′-OH); 7.63 (*d*, 1H, H-6′); 7.32 (*m*, 2H, H-2 and H-6); 3.04 (*m*, 1H, H-7);7.24 (*m*, 3H, H-3, H-4 and H-5); 6.37 (*m*, 2H, H-3′and H-5′), 3.23 (*m*, 1H, H-8). ^{13}C NMR (100 MHz, $CDCl_3$): δ 203.7 (C-9); 165.1 (C-4′); 162.7 (C-2′); 39.7 (C-8); 140.8 (C-1); 132.2 (C-6′); 126.3 (C-4);128.6 (C-2); 128.4 (C-6); 128.4 (C-3); 128.6 (C-5); 30.3 (C-7); 113.7 (C-1′); 107.9 (C-5′); 103.5 (C-3′).

1-[2,4-dihydroxy-3-(3-methylbut-2-en-1-yl)phenyl]-3-phenylpropan-1-one (**2**): Colorless oil with a yield of 2.63 %. ^{1}H ($CDCl_3$, 400 MHz) *d* 7.30 (2H, *m*, H-2/H-6), 7.25 (3H, *m*, H-3,4,5), 6.35 (1H, *d*, *J*= 8.8, H-5′), 7.54 (1H, *d*, *J*= 8.8, H-6′), 3.44 (2H, *da*, *J*= 7.1, H-1′′), 5.26 (1H, *tt*, *J*= 1.3, 6.1, H-2′′), 1.76 (3H, *s*, CH_3), 1.82 (3H, *s*, CH_3), 3.24 (2H, *t*, *J*= 7.7, Ha), 3.04 (2H, *t*, *J*= 7.7, Hb), 6.06 (1H, *s*, OH-4′), 13.14 (1H, *s*, OH-2′); ^{13}C ($CDCl_3$, 100MHz) *d* 141.1 (C-1), 128.5 (C-2/C-6), 128.7 (C-3/C-5), 126.4 (C-4), 113.4 (C-1′), 162.8 (C-2′), 114.1 (C-3′), 161.5 (C-4′), 107.9 (C-5′), 129.6 (C-6′), 21.8 (C-1′′), 121.1 (C-2′′), 136.1 (C-3′′), 26.0 (CH_3), 18.1 (CH_3), 39.8 (C*a*), 30.6 (C*b*), 203.9 (C=O).

(2*E*)-1-(2,4-dihydroxyphenyl)-3-phenylprop-2-en-1-one (**3**): It was obtained as a yellow powder with a yield of 8.7 %.^{1}H (400 MHz, $CDCl_3$): δ 13.38 (1H, 2′-OH); 7.91 (*s*, 1H, H-8); 7.92 (*m*, 1H, H-6′); 7.65 (*m*, 2H, H-2 and H-6); 7.55 (*s*, 1H, H-7);7.43 (*m*, 3H, H-3, H-4 and H-5); 6.45 (*m*, 2H, H-3′and H-5′); 5,92 (1H, 4′-OH). ^{13}C (100 MHz, $CDCl_3$): δ 192.0 (C-9); 166.4 (C-4′); 162.7 (C-2′); 144.6 (C-8); 134.7 (C-1); 132.0 (C-6′); 130.7 (C-4);129.0 (C-2); 129.0 (C-6); 128.5 (C-3); 128.7 (C-5); 120.2 (C-7); 114.5 (C-1′); 107.8 (C-5′); 103.8 (C-3′).

2′, 4′-Dihydroxy-3′-(3-methylbut-2-enyl)chalcone (**4**): Yellow amorphous solid with a yield of 3.95 %. ^{1}H (500 MHz, $CDCl_3$): ^{1}H (500 MHz, $CDCl_3$): *δ* 1.78 (3H, *s*, 3″-Me), 1.85 (3H, *s*, 3″-Me), 3.49 (2H, *d*, *J*=7.0 Hz, H-1″), 5.31 (1H, *t*, *J*=7.0 Hz, H-2″), 6.44 (1H, *d*, *J*=8.9 Hz, H-5′), 7.43 (5H, *m*, B ring protons), 7.60 (1H, *d*, *J*=15.4 Hz, Hα), 7.74 (1H, *d*, *J*=8.9 Hz, H-6′), 7.89 (1H, *d*, *J*=15.4 Hz, Hβ), 13.76 (1H, *s*, OH). ^{13}C (125 MHz, $CDCl_3$): *δ* 18.2 (3″-Me), 22.0 (C-1″), 26.0 (3″-Me), 108.1 (C-5′), 114.3 (C-1′), 114.4 (C-3′), 120.8 (C-*α*), 121.3 (C-2″), 128.7 (C-2/C-6), 129.2 (C-3/C-5), 129.6 (C-6′), 130.8 (C-4), 135.1 (C-1) 136.1 (C-3″), 144.5 (C-β), 162.0 (C-4′), 164.2 (C-2′), 192.3 (C=O).

(2*E*)-1-(2,4-dihydroxyphenyl)-3-phenylprop-2-en-1-one (**5**): It was obtained as a yellow powder with a yield of 3.0 %. ^{1}H NRM (400 MHz, $CDCl_3$): δ 13.71 (*s*, 1H, 2′-OH); 7,87 (*s*, 1H, H-6′); 7.74 (*d*, 1H, H-8); 7.65 (*m*, 2H, H-2 and H-6); 7.60 (*d*, 1H, H-7); 7.43 (*m*, 3H, H-3, H-4 and H-5); 6.42 (*s*, 1H, H-3′); 5.30 (*t*, 1H, H-8′); 3.08 (*d*, 1H, H-7′); 1.58 (*s*, 1H, H-10′); 1.52 (*s*, 1H, H-11′). ^{13}C NRM (100 MHz, $CDCl_3$): δ 191.6 (C-9); 163.9 (C-2′); 161.7 (C-4′); 144.2 (C-7); 136.0 (C-9′); 134.8 (C-1); 130.6 (C-4); 129.3 (C-6′); 129.0 (C-2); 129.0 (C-6); 128.5 (C-3); 128.5 (C-5); 121.0 (C-8′); 120.5 (C-8); 114.5 (C-1′); 114.1 (C-3′); 107.9 (C-5′); 28.8 (C-7′); 25.8 (C-10′); 17.8 (C-11′).

S2. Spectroscopic data and structures of known dihydrochalcones and chalcones derivatives (6-29).

1-[4-(allyloxy)-2-hydroxyphenyl]-3-phenylpropan-1-one (**6**): The compound was isolated as a yellow oil with a yield of 76.3%. ^{1}H NMR (400 MHz, $CDCl_3$): δ 12.78 (*s*, 1H, 2′-OH), 7.64 (*m*, 1H, H-6′); 7.31 (*m*, 2H, H-2 and H-6); 7.25 (*m*, 3H, H-3, H-4 and H-5); 6.42 (*m*, 2H, H-3′ and H-5′); 5.87 (*m*, 2H, H-2 and H-3′′a); 5.72 (*m*, 1H, H-3′′b); 4.48 (*d*, *J* =6.0 Hz, 2H, H-1′′); 3.23 (*m*, 2H, H-8), 3.05(*m*, 2H, H-7). ^{13}C NMR (100 MHz, $CDCl_3$): δ 203.3 (C-9); 165.3 (C-2′); 165.1 (C-4′); 140.9 (C-1); 131.5 (C-2′′); 131.4 (C-6′); 128.6 (C-2 and C-6); 128.5 (C-3 and C-5); 126.3 (C-4); 118.4 (C-3′′); 113.4 (C-1′); 108.5 (C-5′); 101.7 (C-3′); 69.0 (C-1′′); 40.2 (C-8); 30.3 (C-7). HRMS: $[M + H]^+$ ion m/z 283.3402 ($C_{18}H_{18}O_3$: 282.3339).

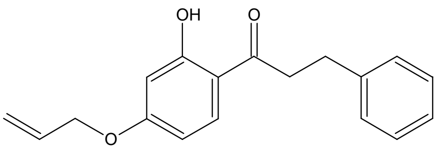

6

1-[4-(crotyloxy)-2-hydroxyphenyl]-3-phenylpropan-1-one (**7**): The compound was isolated as a light brown oil with a yield of 73.4%. ^{1}H NMR (400 MHz, $CDCl_3$): δ 12.78 (*s*, 1H, 2′-OH), 7.63 (*m*, 1H, H-6′); 7.30 (*m*, 2H, H-2 and H-6); 7.23 (*m*, 3H, H-3, H-4 and H-5); 6.42 (*m*, 2H, H-3′ and H-5′); 5.85 (*m*, 1H, H-2′′); 5.71 (*m*, 1H, H-3′′); 4.48 (*d*, *J* =6.1 Hz, 2H, H-1′′); 3.24 (*m*, 2H, H-8), 3.05(*m*, 2H, H-7); 1.77 (*s*, 3H, H-4′′). ^{13}C NMR (100 MHz, $CDCl_3$): δ 203.5 (C-9); 165.3 (C-2′); 165.2 (C-4′); 140.9 (C-1); 131.5 (C-2′′); 131.4 (C-6′); 128.6 (C-2 and C-6); 128.4 (C-3 and C-5); 126.3 (C-4);125.0 (C-1′); 113.4 (C-3′′); 108.1 (C-5′); 101.7 (C-3′); 69.0 (C-1′′); 39.6 (C-8); 30.3 (C-7); 17.9 (C-4′′). HRMS: $[M + H]^+$ ion m/z 297.3671 ($C_{19}H_{20}O_3$: 296.3603).

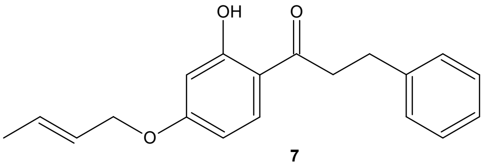

7

1-[4-(2-methylpropenyloxy)-2-hydroxyphenyl]-3-phenylpropan-1-one (**8**): Compound was isolated as a light-yellow oil with a yield of 74.0 %. ^{1}H NMR (400 MHz, $CDCl_3$): δ 12.76 (*s*, 1H, 2′-OH), 7.63 (*m*, 1H, H-6′); 7.31 (*m*, 2H, H-2 and H-6); 7.23 (*m*, 3H, H-3, H-4 and H-5); 5.08 (*m*, 1H, H-3′′b); 5.01 (*m*, 1H, H-3′′a); 4.46 (*s*, 2H, H-1′′); 3.24 (*m*, 2H, H-8), 3.05(*m*, 2H, H-7); 1.82 (*s*, 3H, H-4′′). ^{13}C NMR (100 MHz, $CDCl_3$): δ 203.5 (C-9); 165.3 (C-2′); 165.2 (C-4′); 140.9 (C-1); 139.8 (C-2′′); 131.4 (C-6′); 128.6 (C-2 and C-6); 128.4 (C-3 and C-5); 126.3 (C-4); 113.5 (C-1′); 113.3 (C-3′′); 108.1 (C-5′); 101.9 (C-3′); 71.9 (C-1′′); 39.7 (C-8); 30.3 (C-7); 19.3 (C-4′′). HRMS: $[M + H]^+$ ion m/z 297.3791 ($C_{19}H_{20}O_3$: 296.3506).

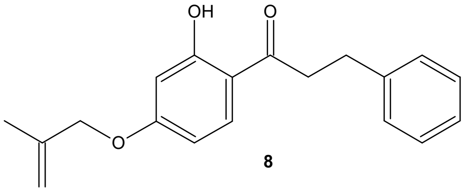

8

1-[4-(prenyloxy)-2-hydroxyphenyl]-3-phenylpropan-1-one (**9**): The compound was isolated as a yellow solid in a yield of 71.1% and a melting point 89-91°C. ^{1}H NMR (400 MHz, $CDCl_3$): δ 12.78 (*s*, 1H, 2′-OH), 7.63 (*d*, *J* =9.4 Hz, 1H, H-6′); 7.31 (*m*, 2H, H-2 and H-6); 7.23 (*m*, 3H, H-3, H-4 and H-5); 6.42 (*m*, 2H, H-3′ and H-5′); 5.47 (*m*, 1H, H-2′′); 4.54 (*d*, *J* =6.7 Hz, 2H, H-1′′); 3.23 (*m*, 2H, H-8), 3.05 (*m*, 2H, H-7); 1.80 (*s*, 3H, H-4′′); 1.74 (*s*, 3H, H-5′′). ^{13}C NMR (100 MHz, $CDCl_3$): δ 203.4 (C-9); 166.9 (C-2′); 165.8 (C-4′); 144.3 (C-1); 138.0 (C-3′′); 130.6 (C-6′); 129.0 (C-2 and C-6); 128.5 (C-3 and C-5); 126.3 (C-4); 120.4 (C-2′′); 114.0 (C-1′); 108.3 (C-5′); 101.7 (C-3′); 65.2 (C-1′′); 40.9 (C-8); 29.4 (C-7); 25.8 (C-5′′); 18.2 (C-4′′). HRMS: $[M + H]^+$ ion m/z 311.3941 ($C_{20}H_{22}O_3$: 310.3869).

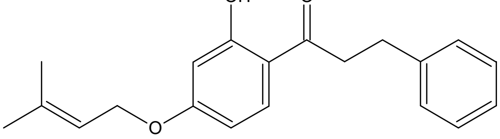

9

1-[4-(geranyloxy)-2-hydroxyphenyl]-3-phenylpropan-1-one (**10**): The compound was isolated as a yellow oil with a yield of 46.6%. ^{1}H NMR (400 MHz, $CDCl_3$): δ 12.79 (*s*, 1H, 2′-OH), 7.63 (*d*, *J* =9.6 Hz, 1H, H-6′); 7.30 (*m*, 2H, H- 2 and H-6); 7.25 (*m*, 3H, H-3, H-4 and H-5); 6.42 (*m*, 2H, H-3′ and H-5′); 5.45 (*m*, 1H, H-2′′); 5.07 (*m*, 1H, H-7′′); 4.56 (*d*, *J* =6.6 Hz, 2H, H-1′′); 3.23 (*m*, 2H, H-8), 3.05(*m*, 2H, H-7); 2.09 (*m*, 4H, H-5′′ and H-6′′), 1.73 (*s*, 3H, H-4′′); 1.67 (*s*, 3H, H-9′′), 1.60 (*s*, 3H, H-10′′). ^{13}C NMR (100 MHz, $CDCl_3$): δ 203.5 (C-9); 165.4 (C-2′ and C-4′); 142.2 (C-1); 140.9 (C-3′′); 131.9 (C-8′′); 131.4 (C-6′); 128.6 (C-2 and C-6); 128.4 (C-3 and C-5); 126.3 (C-4); 123.7 (C-7′′); 118.2 (C-2′′); 113.3 (C-1′); 108.2 (C-5′); 101.7 (C-3′); 65.2 (C-1′′); 39.6 (C-8); 39.5 (C-5′′); 30.4 (C-7); 26.2 (C-6′′); 25.6 (C-10′′); 17.7 (C-9′′); 16.7 (C-4′′). HRMS: $[M + H]^+$ ion m/z 379.5131 ($C_{25}H_{30}O_3$: 378.5038).

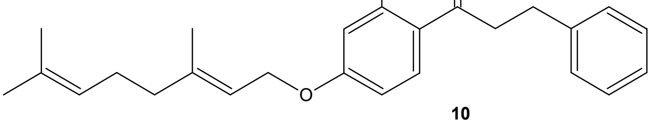

10

1-[4-(farnecyloxy)-2-hydroxyphenyl]-3-phenylpropan-1-one (**11**): The compound was isolated as a pale-yellow oil with a yield of 33.3%. ^{1}H NMR (400 MHz, $CDCl_3$): 7.87 (*d*, *J* =8.8 Hz, 1H, H-6′); 7.48 (*m*, 2H, H-2 and H-6); 7.38 (*m*, 3H, H-3, H-4 and H-5); 6.62 (*d*, *J* =8.8 Hz, 2H, H-5′ and H-3′); 5.44 (*m*, 1H, H-2′′); 5.09 (*m*, 2H, H-7′′ and H-12′′); 4.62 (*d*, *J* =6.3 Hz, 2H, H-1′′); 3.00 (*m*, 2H, H-8); 2.86 (*m*, 2H, H-7); 2.11 (*m*, 4H, H-5′′ and H-6′′), 2.06 (*m*, 4H, H-10′′ and H-11′′), 1.79 (*s*, 3H, H-4′′); 1.73 (*s*, 3H, H-10′′); 1.67 (*s*, 6H, H-9′′ and H-14′′). ^{13}C NMR (100 MHz, $CDCl_3$): δ 201.5 (C-9); 163.7 (C-2′); 160.1 (C-4′); 142.0 (C-4′′); 141.3 (C-1); 135.8 (C-8′′); 131.3 (C-13′′); 129.7 (C-6′); 128.7 (C-2 and C-6); 128.2 (C-3

and C-5); 127.7 (C-4); 124.3 (C-7′′); 123.5 (C-12′′); 118.9 (C-2′′); 118.7 (C-1′); 114.1 (C-1′); 106.1 (C- 5′); 100.3 (C-3′); 65.1 (C-1′′); 39.6 (C-8); 39.5 (C-5′′ and C-10′′); 30.2 (C-7); 26.7 (C-11′′); 26.2 (C-6′′); 25.7 (C-15′′); 17.7 (C-14′′); 16.8 (C-4′′); 16.0 (C-9′′). HRMS: $[M + H]^+$ ion m/z 447.6321 ($C_{30}H_{36}O_3$: 446.6209).

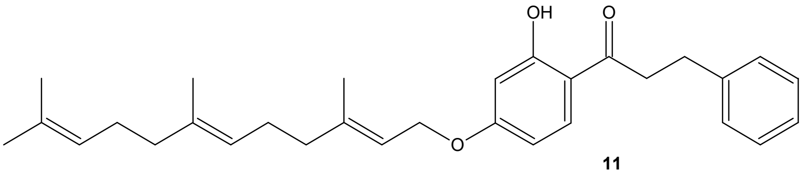

11

1-[4-(allyloxy)-2-hydroxy-3-(prenyl)phenyl]-3-phenylpropan-1-one (**12**): Compound was isolated as a pale oil in a yield of 89.3%. ^{1}H NMR (400 MHz, $CDCl_3$): δ 12.58 (*s*,1H, 2′-OH), 7.44 (*s*, 1H, H-6′); 7.27 (*m*, 5H, H-2, H-3, H-4, H-5 y H-6); 6.37 (*s*,1H, H-5′); 6.01 (*m*, 1H, H-2′′); 5.44 (*d*, *J*=17.2 Hz,1H, H 3′′b); 5.44 (*d*, *J* =10.6 Hz,1H, H-3′′a); 5.16 (*m*, 1H, H-8′); 4.43 (*d*, *J* =6.6 Hz, 2H, H-1′′); 3.25 (*m*, 2H, H-8), 3.22 (*m*, 2H, H-7′); 3.05(*m*, 2H, H-7); 1.78 (*s*, 3H, H-10′); 1.76 (*s*, 3H, H-11′). ^{13}C NMR (100 MHz, $CDCl_3$): δ 203.8 (C-9); 163.7 (C-2′); 161.4 (C-4′); 141.1 (C-1); 133.3 (C-2′′); 131.4 (C-6′); 128.6 (C-2 and C-6); 128.4 (C-3 and C-5); 126.3 (C-4); 121.4 (C-8′); 118.9 (C-1′); 117.2 (C-3′′); 113.6 (C-1′); 103.9 (C-5′); 69.6 (C-1′′); 39.8 (C-8); 30.5 (C-7); 25.8 (C-7′); 21.0 (C-7′); 17.9 (C-10′). HRMS: $[M + H]^+$ ion m/z 335.4598 ($C_{23}H_{26}O_3$: 334.4510).

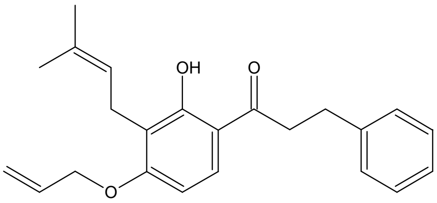

12

1-[4-(crotyloxy)-2-hydroxy-3-(prenyl)phenyl]-3-phenylpropan-1-one (**13**): Compound was isolated as a yellow oil with a yield of 88.0%. ^{1}H NMR (400 MHz, $CDCl_3$): δ 12.58 (*s*,1H, 2′-OH), 7.44 (*s*, 1H, H-6′); 7.27 (*m*, 5H, H-2, H-3, H-4, H-5 and H-6); 6.37 (*s*,1H, H-5′); 5.91 (*m*, 2H, H-2′′ and H-3′′); 5.74 (*m*, 1H, H-8′); 4.51 (*d*, *J* =6.0 Hz, 2H, H-1′′); 3.26 (*m*, 2H, H-8), 3.22 (*m*, 2H, H-7′); 3.05(*m*, 2H, H-7); 1.80 (*s*, 3H, H-10′); 1.76 (*s*, 3H, H-11′); 1.68 (*s*, 3H, H-4′′). ^{13}C NMR (100 MHz, $CDCl_3$): δ 203.5 (C-9); 163.2 (C-2′); 162.6 (C-4′); 141.2 (C-1); 131.7 (C-9′); 130.5 (C-6′); 130.3 (C 2′′); 129.0 (C-2 and C-6); 128.5 (C-3 and C-5); 125.6 (C-4); 122.0 (C-8′); 118.2 (C- 3′); 103.3 (C-5′); 69.2 (C-1′′); 40.0 (C-8); 30.1 (C-7); 25.8 (C-11′); 21.8 (C-7′); 17.9 (C-10′); 17.8 (C-4′′). HRMS: $[M + H]^+$ ion m/z 365.4861 ($C_{24}H_{28}O_3$: 364.4774).

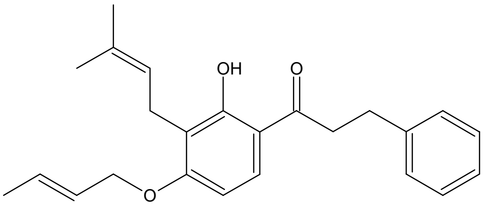

13

1-[4-(2-methylpropenyloxy)-2-hydroxy-3-(prenyl)phenyl]-3-phenylpropan-1-one (**14**): Compound was isolated as a light yellow oil in a yield of 75.0%. ^{1}H NMR (400 MHz, $CDCl_3$): δ 12.58 (*s*, 1H, 2′-OH), 7.63 (*d*, *J* =8.7 Hz, 1H, H-6′); 7.27 (*m*, 5H, H-2, H-3, H-4, H-5 and H-6); 6.45 (*m*, 1H, H-5′); 5.30 (*m*, 1H, H-8′); 5.07 (*m*, 1H, H-3′′b); 5.01(*m*, 1H, H-3′′a); 4.46 (*s*, 2H, H-1′′); 3.19 (*d*, *J* =8.3 Hz, 2H, H-7′); 3.10 (*m*, 2H, H-8); 2.80 (*m*, 2H, H-7); 1.82 (*s*, 6H, H-10′ and H-4′′); 1.65 (*s*, 3H, H-11′). ^{13}C NMR (100 MHz, $CDCl_3$): δ 203.5 (C-9); 163.2 (C-2′); 160.3 (C-4′); 141.0 (C-1); 140.3 (C-2′′); 131.8 (C-9′); 130.5 (C-6′); 129.0 (C-2 and C-6); 128.5 (C-3 and C-5); 126.1 (C-4); 122.0 (C-8′); 117.8 (C-3′′); 114.6 (C-1′); 112.9 (C-3′′); 71.9 (C-1′′); 39.1 (C-8); 31.0 (C-7); 25.8 (C-11′); 21.8 (C-7′); 19.3 (C-4′′); 17.9 (C-10′). HRMS: $[M + H]^+$ ion m/z 365.4863 ($C_{19}H_{20}O_3$: 364.4772).

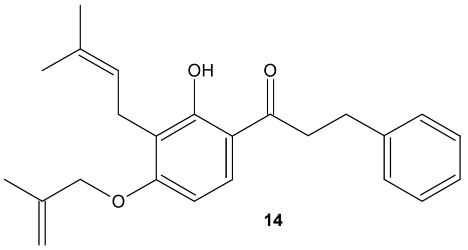

14

1-[4-(prenyloxy)-2-hydroxy-3-(prenyl)phenyl]-3-phenylpropan-1-one (**15**): Compound was isolated as a white solid with a yield of 89.1% and a melting point of 92-93 °C. ^{1}H NMR (400 MHz, $CDCl_3$): δ 12.61 (*s*, 1H, 2′-OH), 7.87 (*d*, *J* =15.4 Hz, 1H, H-6′); 7.27 (*m*, 5H, H-2, H-3, H-4, H-5 and H-6); 6.49 (*d*, *J* =9.0 Hz, 1H, H-5′); 5.47 (*m*, 1H, H-2′′); 5.25 (*m*, 1H, H-8′); 4.63 (*d*, *J* =6.6 Hz, 2H, H-1′′); 3.22 (*m*, 2H, H-7′); 3.10 (*d*, *J* =7.2 Hz, 2H, H-8); 2.82 (*m*, 2H, H-7); 1.79 (*s*, 6H, H-10′ and H-4′′); 1.75 (*s*, 3H, H-5′′); 1.68 (*s*, 6H, H-11′). ^{13}C NMR (100 MHz, $CDCl_3$): δ 203.5 (C-9); 163.1 (C-2′); 162.8 (C-4′); 141.6 (C-1); 136.0 (C-3′′); 131.6 (C-9′); 130.5 (C-6′); 129.0 (C-2 and C-6); 128.5 (C-3 and C-5); 126.1 (C-4); 122.7 (C-8′); 119.5 (C-2′′); 117.9 (C-3′); 114.6 (C-1′); 103.3 (C-5′); 65.3 (C-1′′); 39.1 (C-8); 30.1 (C-7); 25.8 (C-4′′); 25.7 (C-11′); 21.8 (C-7′); 18.3 (C-5′′); 17.8 (C-10′).HRMS: $[M + H]^+$ ion m/z 379.5132 ($C_{25}H_{30}O_3$: 378.5038).

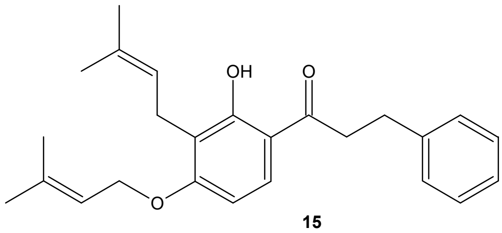

15

1-[4-(geranyloxy)-2-hydroxy-3-(prenyl)phenyl]-3-phenylpropan-1-one (**16**): Compound was isolated as a pale yellow oil with a yield of 73.6 %. ^{1}H NMR (400 MHz, $CDCl_3$): δ 12.60 (*s*, 1H, 2′-OH), 7.87 (*d*, *J* =15.5 Hz, 1H, H-6′); 7.27 (*m*, 5H, H-2, H-3, H-4, H-5 and H-6); 6.49 (*d*, *J* =9.0 Hz, 1H, H-5′); 5.48 (*m*, 1H, H-2′′); 5.25 (*m*, 1H, H-8′); 5.08 (*m*, 1H, H-6′′); 4.65 (*d*, *J* =6.4 Hz, 2H, H-1′′); 3.22 (*m*, 2H, H-8), 3.10 (*d*, *J* =7.2 Hz, 2H, H-8); 2.82 (*m*, 2H, H-7); 2.09 (*m*, 4H, H-4′′ and H-5′′), 1.79 (*s*, 3H, H-10′); 1.75 (*s*, 3H, H-9′′); 1.67 (*s*, 6H, H-11′ and H-8′′); 1.61 (*s*, 3H, H-10′′). ^{13}C NMR (100 MHz, $CDCl_3$): δ 203.5 (C-9);

163.2 (C-2′); 159.5 (C-4′); 141.3 (C-1 and C-3″); 131.6 (C-9′ and C-7″); 130.2 (C-6′); 129.0 (C-2 and C-6); 128.5 (C-3 and C-5); 126.3 (C-4); 123.7 (C-6″); 122.1 (C-8″); 119.3 (C-2″); 117.8 (C-3′); 114.4 (C-1′); 103.3 (C-5′); 65.4 (C-1″); 43.9 (C-8); 39.5 (C-4″); 30.9 (C-7); 26.3 (C-5″); 25.8 (C-11″); 25.7 (C-8″); 21.8 (C-7′); 17.8 (C-10′); 17.7 (C-10″); 16.7 (C-9″). HRMS: $[M + H]^+$ ion m/z 447.6321 ($C_{30}H_{38}O_3$: 446.6207).

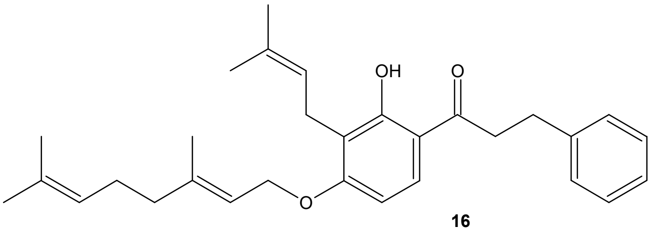

16

1-[4-(farnecyloxy)-2-hydroxy-3-(prenyl)phenyl]-3-phenylpropan-1-one (**17**): Compound was isolated as a pale yellow oil in a yield of 56.6%. ^{1}H NMR (400 MHz, $CDCl_3$): δ 12.58 (*s*, 1H, 2′-OH), 7.87 (*d*, *J* =15,5 Hz, 1H, H-6′); 7.28 (*m*, 5H, H-2, H-3, H-4, H-5 and H-6); 6.49 (*d*, *J* =9.0 Hz, 1H, H-5′); 5.48 (*m*, 1H, H-2″); 5.25 (*m*, 1H, H-8′); 5.08 (*m*, 2H, H-6″ and H-10″); 4.65 (*d*, *J* =6.6 Hz, 2H, H-1″); 3.22 (*d*, *J* =9.0 Hz, 2H, H-7′); 3.10 (*m*, 2H, H-8); 2.82 (*m*, 2H, H-7); 2.07 (*m*, 4H, H-4″, H-5″, H-8″ and H- 9″); 1.79 (*s*, 6H, H-10′ and H-13″); 1.75 (*s*, 3H, H-11′); 1.67 (*s*, 6H, H-14″ and H-15″). ^{13}C NMR (100 MHz, $CDCl_3$): δ 203.5 (C-9); 163.2 (C-2′); 159.6 (C-4′); 141.7 (C-1y C-3″); 131.3 (C-9′ and C-11″); 130.5 (C-6′); 128.9 (C-2 and C-6); 128.5 (C-3 and C-5); 126.7 (C-4); 124.3 (C-6″); 122.1 (C-8′); 119.3 (C-2″); 118.7 (C-3′); 114.5 (C-1′); 103.3 (C-5′); 65.8 (C-1″); 44.1 (C-8); 39.7 (C-4″); 39.4 (C-8″); 30.2 (C-7); 26.7 (C-9″); 26.2 (C-5″); 25.8 (C-12″); 25.7 (C-11′); 21.8 (C-7′); 17.9 (C-10′); 17.7 (C-15″); 16.7 (C-13″); 16.0 (C-14″). HRMS: $[M + H]^+$ ion m/z 515.7512 ($C_{35}H_{46}O_3$: 514.7380).

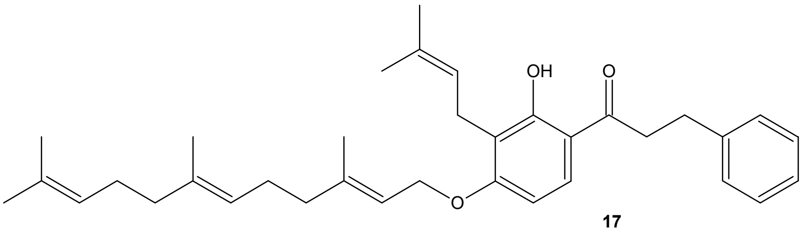

17

(2*E*)-1-[4-(allyloxy)-2-hydroxyphenyl]-3-phenylprop-2-en-1-one (**18**): Compound was isolated as an orange solid in a yield of 75.0% and a melting point of 109 °C. ^{1}H NMR (400 MHz, $CDCl_3$): δ 13.41 (*s*, 1H, 2′-OH), 7.91 (*s*, 1H, H-7); 7.85 (*m*, 1H, H-6′); 7.65 (*m*, 2H, H-2 and H-6); 7.57 (*s*, 1H, H-8); 7.43 (*m*, 3H, H-3, H-4 and H-5); 6.51 (*m*, 1H, H-5′); 6.48 (*s*, 1H, H-3′); 6.05 (*m*, 1H, H- 2″); 5.44 (*d*, *J* =17.2 Hz, 1H, H-3″a); 5.33 (*d*, *J* =10.6 Hz, 1H, H-3″b); 4.60 (*d*, *J* =5.3 Hz, 2H, H-1″). ^{13}C NMR (100 MHz, $CDCl_3$): δ 191.9 (C-9); 166.6 (C-2′); 165.2 (C-4′); 144.4 (C-7); 134.8 (C-1); 132.2 (C-2″); 131.3 (C-6′); 130.7 (C-4); 129.0 (C-3 and C-5); 128.5 (C-2 and C-6); 124.0 (C-1′); 120.3 (C-8); 118.4 (C-3″); 108.2 (C-5′); 101.9 (C-3′); 69.0 (C-1″). HRMS: $[M + H]^+$ ion m/z 281.3242 ($C_{18}H_{16}O_3$: 280.3178).

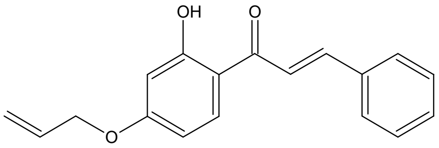

18

(2*E*)-1-[4-(crotyloxy)-2-hydroxyphenyl]-3-phenylprop-2-en-1-one (**19**): Compound was isolated as an orange solid in a yield of 72.0% and a melting point of 110 °C. ^{1}H NMR (400 MHz, $CDCl_3$): δ 13.43 (*s*, 1H, 2′-OH), 7.91 (*s*, 1H, H-7); 7.84 (*m*, 1H, H-6′); 7.64 (*m*, 2H, H-2 and H-6); 7.57 (*s*, 1H, H-8); 7.43 (*m*, 3H, H-3, H-4 and H-5); 6.49 (*m*, 2H, H-3′ and H-5′); 5.93 (*m*, 1H, H-2″); 5.73 (*m*, 1H, H-3″); 4.51 (*d*, *J* =6.1 Hz, 2H, H-1″); 1.77 (*s*, 3H, H-4″). ^{13}C NMR (100 MHz, $CDCl_3$): δ 191.8 (C-9); 166.6 (C-2′); 165.4 (C-4′); 144.8 (C-7); 134.8 (C-1); 131.6 (C-2″); 131.1 (C-6′); 130.6 (C-4); 129.0 (C-3 and C-5); 128.5 (C-2 and C-6); 125.0 (C-1′); 120.4 (C-8); 114.1 (C-3″); 108.3 (C-5′); 101.8 (C-3′); 69.0 (C-1″); 17.9 (C-4″). HRMS: $[M + H]^+$ ion m/z 295.3512 ($C_{19}H_{18}O_3$: 294.3444).

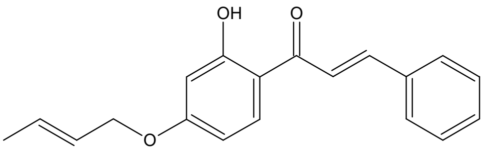

19

(2*E*)-1-[4-(2-methylpropenyloxy)-2-hydroxyphenyl]-3-phenylprop-2-en-1-one (**20**): The compound was isolated as an orange solid in a yield of 72.0 % and a melting point of 108-110 °C. ^{1}H NMR (400 MHz, $CDCl_3$): δ 13.40 (s, 1H, 2′-OH), 7.91 (s, 1H, H-7); 7.84 (m, 1H, H-6′); 7.64 (*m*, 2H, H-2 and H-6); 7.57 (*s*, 1H, H-8); 7.43 (*m*, 3H, H-3, H-4 and H-5); 6.51 (*m*, 2H, H-3′ and H-5′); 5.10 (*s*, 1H, H-3″b); 5.03 (*s*, 1H, H-3″a); 4.47 (*d*, *J* =11.12 Hz, 2H, H-1″); 1.84 (*s*, 3H, H-4″). ^{13}C NMR (100 MHz, $CDCl_3$): δ 191.8 (C-9); 166.6 (C-2′); 165.4 (C-4′); 144.8 (C-7); 139.9 (C-2″); 134.8 (C-1); 131.2 (C-6′); 130.6 (C-4); 129.0 (C-3 and C-5); 128.7 (C-2 and C-6); 122.4 (C-1′); 120.4 (C-8); 113.4 (C-3″); 108.2 (C-5′); 102.0 (C-3′); 71.9 (C-1″); 19.3 (C-4″). HRMS: $[M + H]^+$ ion m/z 295.3565 ($C_{19}H_{18}O_3$: 294.3335).

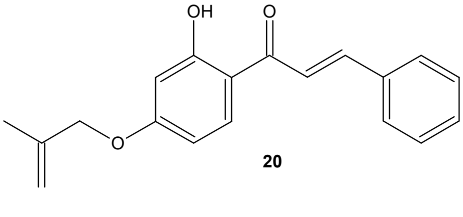

20

(2*E*)-1-[4-(prenyloxy)-2-hydroxyphenyl]-3-phenylprop-2-en-1-one **(21)**: Compound was isolated as a yellow solid with a yield of 69.0% and a melting point of 112-113°C. ^{1}H NMR (400 MHz, $CDCl_3$): δ 13.49 (*s*, 1H, 2′-OH), 7.91 (*s*, 1H, H-7); 7.84 (*d*, *J* =9.6 Hz, 1H, H-6′); 7.30 (*m*, 2H, H-2 and H-6); 7.57 (*s*, 1H, H-8); 7.42 (*m*, 3H, H-3, H-4 and H-5); 6.51 (*m*, 2H, H-3′ and H-5′); 5.49 (*m*, 1H, H-2″); 4.57 (*d*, *J* =6.7 Hz, 2H, H-1″); 1.81 (*s*, 3H, H-4″); 1.79 (*s*, 3H, H-5″). ^{13}C NMR (100 MHz, $CDCl_3$): δ 191.5 (C-9); 166.7 (C-2′); 165.6 (C-4′); 144.3 (C-7); 139.2 (C-3″); 134.9 (C-1); 131.2 (C-6′); 130.6 (C-4); 129.0 (C-3 and

C-5); 128.5 (C-2 and C-6); 120.4 (C-8); 118.7 (C-2′′); 114.0 (C-1′); 108.3 (C-5′); 101.7 (C-3′); 64.6 (C-1′′); 25.8 (C-5′′); 18.2 (C-4′′). HRMS: $[M + H]^+$ ion m/z 309.3782 ($C_{20}H_{20}O_3$: 318.3710).

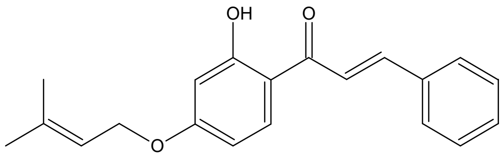

21

(2*E*)-1-[4-(geranyloxy)-2-hydroxyphenyl]-3-phenylprop-2-en-1-one (**22***):* Compound was isolated as a light orange oil with a yield of 47.4%. ^{1}H NMR (400 MHz, $CDCl_3$): δ 13.43 (*s*, 1H, 2′-OH), 7.91 (*s*, 1H, H-7); 7.84(*d*, *J* =9.6 Hz, 1H, H-6′); 7.30 (*m*, 2H, H-2 and H-6); 7.57 (*s*, 1H, H-8); 7.42 (*m*, 3H, H-3, H-4 and H-5); 6.50 (*m*, 2H, H-3′ and H-5′); 5.48 (*m*, 1H, H-2′′); 5.09 (*m*, 1H, H-7′′); 4.60 (*d*, *J* =6.6 Hz, 2H, H-1′′); 2.12 (*m*, 4H, H-5′′ and H-6′′), 1.75 (*s*, 3H, H-4′′); 1.68 (*s*, 3H, H-9′′), 1.61 (*s*, 3H, H-10′′). ^{13}C NMR (100 MHz, $CDCl_3$): δ 191.8 (C-9); 166.7 (C-2′); 166.0 (C-4′); 144.3 (C-7); 141.2 (C-3′′); 134.9 (C-1); 131.2 (C-6′ and C-8′′); 130.6 (C-4); 129.0 (C-3 and C-5); 128.5 (C-2 and C-6); 123.7 (C-7′′); 120.4 (C-8); 118.5 (C-2′′); 114.0 (C-1′); 108.3 (C-5′); 101.8 (C-3′); 65.3 (C-1′′); 39.5 (C- 5′′); 26.3 (C-6′′); 25.6 (C-10′′); 17.7 (C-9′′); 16.7 (C-4′′). HRMS: $[M + H]^+$ ion m/z 377.5221 ($C_{25}H_{28}O_3$: 376.5130).

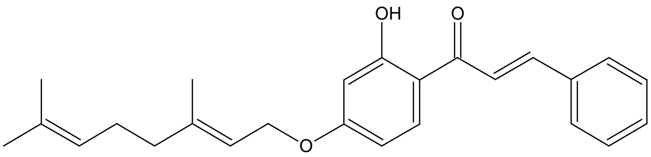

22

(2*E*)-1-[4-(farnesyloxy)-2-hydroxyphenyl]-3-phenylprop-2-en-1-one (**23**): Compound was isolated as a light-yellow oil with a yield of 30.6%. ^{1}H NMR (400 MHz, $CDCl_3$): δ 7.85 (*d*, *J* =9.6 Hz, 1H, H-6′); 7.70 (*s*, 1H, H-7); 7.69 (*s*, 1H, H-8); 7.58 (*m*, 2H, H-2 and H-6); 7.35 (*m*, 3H, H-3, H-4 and H-5); 6.56 (*m*, 1H, H-5′); 6.52 (*m*, 1H, H-3′); 5.50 (*m*, 1H, H-2′′); 5.10 (*m*, 2H, H-7′′ and H-12′′); 4.60 (*d*, *J* =6.6 Hz, 2H, H-1′′); 2.07 (*m*, 4H, H-5′′ and H-6′′), 1.99 (*m*, 4H, H-10′′ and H-11′′), 1.76 (*s*, 3H, H-4′′); 1.73 (*s*, 3H, H-10′′); 1.67 (*s*, 6H, H-9′′ and H-14′′). ^{13}C NMR (100 MHz, $CDCl_3$): δ 189.9 (C-9); 163.7 (C-2′); 160.1 (C-4′); 142.0 (C-7); 142.0 (C-3′′); 135.8 (C-8′′); 135.6 (C-1); 133.1 (C-6′); 131.3 (C-13′′); 129.7 (C-4); 128.7 (C-3 and C-5); 128.2 (C-2 and C-6); 124.3 (C-7′′); 123.6 (C-12′′); 122.1 (C-8); 118.9 (C-2′′); 118.7 (C-1′); 106.1 (C-5′); 100.3 (C-3′); 65.6 (C-1′′); 39.7 (C-5′′); 39.6 (C-10′′); 26.2 (C-11′′ and C-6′′); 25.7 (C-15′′); 17.7 (C-14′′); 16.7 (C-4′′); 16.0 (C-9′′). HRMS: $[M + H]^+$ ion m/z 445.6162 ($C_{30}H_{36}O_3$: 444.605).

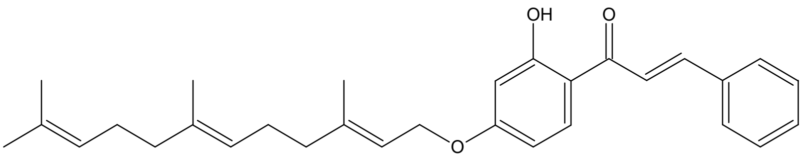

23

(2*E*)-1-[4-(allyloxy)-2-hydroxy-3-(prenyl)phenyl]-3-phenylprop-2-en-1-one (**24**): The compound was isolated as a yellow solid in a yield of 85.0% and a melting point of 96 °C. ^{1}H NMR (400 MHz, $CDCl_3$): δ 13.37 (*s*, 1H, 2′-OH), 7.88 (*d*, *J*=15.4 Hz, 1H, H-6′); 7.78 (*d*, *J* =9.1 Hz, 1H, H-8); 7.62 (*m*, 2H, H-7 and H-4); 7.42 (*m*, 4H, H-2, H-3, H-5 and H-6); 6.48 (*d*, *J* =9.0 Hz, 1H, H-5′); 6.07 (*m*, 1H, H- 2′′); 5.45 (*m*, 1H, H-3′′b); 5.31 (*m*, 1H, H-3′′a); 5.27 (*m*, 1H, H-8′); 4.65 (*d*, *J* =5.1 Hz, 2H, H-1′′); 3.42 (*d*, *J* =7.1 Hz, 1H, H-7′); 1.80 (*s*, 3H, H-10′); 1.68(*s*, 3H, H- 11′). ^{13}C NMR (100 MHz, $CDCl_3$): δ 192.4 (C-9); 163.2 (C-2′); 162.4 (C-4′); 144.1 (C-7); 132.7 (C-1); 131.8 (C-9′); 130.5 (C-6′); 129.1 (C-4); 129.0 (C-2 and C-6); 128.5 (C-3 and C-5); 121.9 (C-8′); 120.6 (C-8); 118.0 (C-3′) 117.6 (C-3′′); 114.6 (C- 1′); 118.4 (C-3′′); 103.2 (C-5′); 69.2 (C-1′′); 25.8 (C-11′); 21.8 (C-7′); 17.9 (C- 10′). HRMS: $[M + H]^+$ ion m/z 349.4432 ($C_{23}H_{24}O_3$: 348.4348).

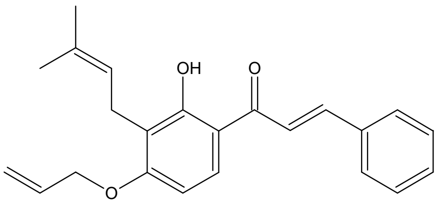

24

(2*E*)-1-[4-(crotyloxy)-2-hydroxy-3-(prenyl)phenyl]-3-phenylprop-2-en-1-one (**25**): The compound was isolated as a yellow solid in a yield of 85.0 % and a melting point of 93-94°C. ^{1}H NMR (400 MHz, $CDCl_3$): δ 13.37 (*s*, 1H, 2′-OH), 7.87 (*d*, *J* =15.5 Hz, 1H, H-6′); 7.77 (*d*, *J* =9.1 Hz, 1H, H-8); 7.63 (*m*, 2H, H-7 and H-4); 7.42 (*m*, 4H, H-2, H-3, H-5 and H-6); 6.49 (*d*, *J* =9,0 Hz, 1H, H-5′); 5.87 (*m*, 1H, H-2′′); 5.82 (*m*, 1H, H-3′′); 5.26 (*m*, 1H, H-8′); 4.57 (*d*, *J* =5.9 Hz, 2H, H-1′′); 3.40 (*d*, *J* =7.2 Hz, 1H, H-7′); 1.80 (s, 3H, H-10′); 1.77 (s, 3H, H-4′′); 1.68(s, 3H, H-11′). ^{13}C NMR (100 MHz, $CDCl_3$): δ 192.2 (C-9); 163.2 (C-2′); 162.6 (C-4′); 144.0 (C-7); 134.9 (C-1); 131.7 (C-6′); 130.5 (C-9′); 130.3 (C-4); 129.1 (C-2′′); 129.0 (C-2 and C-6); 128.5 (C-3 and C-5); 125.6 (C-3′′); 122.0 (C-8′); 120.7 (C-8); 118.0 (C-3′) 117.8 (C-3′); 115.6 (C-1′); 103.3 (C-5′); 69.0 (C-1′′); 25.8 (C-11′); 21.8 (C-7′); 17.9 (C-10′); 17.8 (C-4′′). HRMS: $[M + H]^+$ ion m/z 363.4702 ($C_{24}H_{26}O_3$: 362.4614).

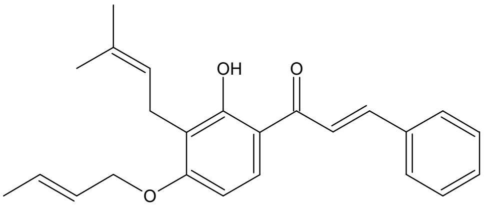

25

(2*E*)-1-[4-(2-methylpropenyloxy)-2-hydroxy-3-(prenyl)phenyl]-3-phenylprop-2-en-1-one (**26**): The compound was isolated as a yellow solid in a yield of 84.5% and a melting point of 101 °C. ^{1}H NMR (400 MHz, $CDCl_3$): δ 13.39 (*s*, 1H, 2′-OH), 7.87 (*d*, *J* =15.5 Hz, 1H, H-6′); 7.77 (*d*, *J* =9.0 Hz, 1H, H-8); 7.63 (*m*, 2H, H-7 and H-4); 7.42 (*m*, 4H, H-2, H-3, H-5 and H-6); 6.48 (*d*, *J* =9.0 Hz, 1H, H-5′); 5.26 (*m*, 1H, H- 2′′); 5.11 (*s*, 1H, H-3b′′); 5.01 (*s*, 1H, H-3a′′); 4.54 (*s*, 2H, H-1′′); 3.43 (*d*, *J* =7.1 Hz, 1H, H-7′); 1.84 (*s*, 3H, H-4′′); 1.80 (*s*, 3H, H-10′); 1.68(*s*, 3H, H-11′). ^{13}C NMR (100 MHz, $CDCl_3$): δ 192.2 (C-9); 163.2 (C-2′); 162.4 (C-4′); 144.1 (C-7); 134.9 (C-1); 131.8 (C-6′); 130.5 (C-9′); 129.1 (C-4); 129.0 (C-2 and C-6);

128.5 (C-3 and C- 5); 122.0 (C-8′); 120.6 (C-8); 117.8 (C-3′) 117.8 (C-3′); 114.6 (C-1′); 113.0 (C- 3′′); 103.2 (C-5′); 71.9 (C-1′′); 25.8 (C-11′); 21.8 (C-7′); 19.3 (C-4′′); 17.9 (C-10′). HRMS: $[M + H]^+$ ion m/z 363.4702 ($C_{25}H_{28}O_3$: 362.4616).

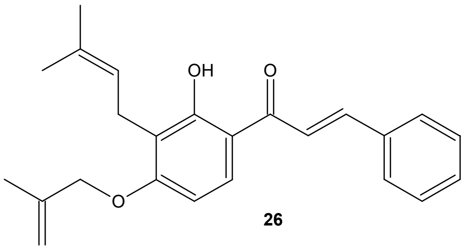

26

(2*E*)-1-[4-(prenyloxy)-2-hydroxy-3-(prenyl)phenyl]-3-phenylprop-2-en-1-one (**27**): The compound was isolated as a yellow solid in a yield of 79.0 % and a melting point of 108 °C. ^{1}H NMR (400 MHz, $CDCl_3$): δ 13.37 (*s*, 1H, 2′-OH), 7.87 (*d*, *J* =15.5 Hz, 1H, H-6′); 7.77 (*d*, *J* =9.1 Hz, 1H, H-8); 7.63 (*m*, 2H, H-7 and H- 4); 7.42 (*m*, 4H, H-2, H-3, H-5 and H-6); 6.49 (*d*, *J* =9.0 Hz, 1H, H-5′); 5.48 (*m*, 1H, H-2′′); 5.25 (*m*, 1H, H-8′); 4.62 (*d*, *J* =6.6 Hz, 2H, H-1′′); 3.39 (*d*, *J* =7.2 Hz, 1H, H- 7′); 1.80 (*s*, 6H, H-4′′ and H-10′); 1.75 (*s*, 3H, H-5′′); 1.68(*s*, 3H, H-11′). ^{13}C NMR (100 MHz, $CDCl_3$): δ 192.1 (C-9); 163.2 (C-2′); 162.8 (C-4′); 144.0 (C-7); 138.1 (C-3′′); 134.9 (C-1); 131.7 (C-6′); 130.5 (C-9′); 129.1 (C-4); 129.0 (C-2 and C-6); 128.5 (C-3 and C-5); 122.1 (C-8′); 120.7 (C-8); 119.5 (C-2′′) 117.9 (C-3′); 114.5 (C- 1′); 103.3 (C-5′); 65.3 (C-1′′); 25.8 (C-4′′); 25.7 (C-11′); 21.8 (C-7′); 18.3 (C-5′′); 17.8 (C-10′). HRMS: $[M + H]^+$ ion m/z 377.4972 ($C_{25}H_{28}O_3$: 376.4880).

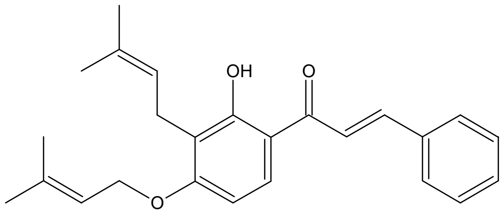

27

(2*E*)-1-[4-(geranyloxy)-2-hydroxy-3-(prenyl)phenyl]-3-phenylprop-2-en-1-one (**28**): The compound was isolated as a yellow solid in a yield of 70.3 % and a melting point of 79 °C. ^{1}H NMR (400 MHz, $CDCl_3$): δ 13.37 (*s*, 1H, 2′-OH), 7.87 (*d*, *J* =15.5 Hz, 1H, H-6′); 7.77 (*d*, *J* =9.1 Hz, 1H, H-8); 7.63 (*m*, 2H, H-7 and H-4); 7.42 (*m*, 4H, H-2, H-3, H-5 and H-6); 6.49 (*d*, *J* =9.0 Hz, 1H, H-5′); 5.48 (*m*, 1H, H-2′′); 5.25 (*m*, 1H, H-8′); 5.08 (*m*, 1H, H-6′′); 4.65 (*d*, *J* =6.4 Hz, 2H, H-1′′); 3.39 (*d*, *J* =7.2 Hz, 1H, H-7′); 2.10 (*m*, 4H, H-4′′ and H-5′′); 1.80 (*s*, 3H, H-10′); 1.75 (*s*, 3H, H-9′′); 1.68 (*s*, 3H, H-8′′); 1.61 (*s*, 3H, H-11′). ^{13}C NMR (100 MHz, $CDCl_3$): δ 191.1 (C-9); 163.1 (C-2′); 162.8 (C-4′); 144.0 (C-7); 141.3 (C-3′′); 134.9 (C-1); 131.9 (C-6′); 131.7 (C-7′′); 130.5 (C-9′); 129.1 (C-4); 129.0 (C-2 and C-6); 128.5 (C-3 and C-5); 123.7 (C-8′); 120.7 (C-8); 119.3 (C-2′′); 117.8 (C-3′); 114.4 (C-1′); 103.3 (C-5′); 65.4 (C-1′′); 39.5 (C-4′′); 26.3 (C-5′′); 25.8 (C-11′); 25.7 (C-8′′); 21.8 (C-7′); 17.9 (C-10′); 17.7 (C-10′′); 16.7 (C-9′′). HRMS: $[M + H]^+$ ion m/z 445.6261 ($C_{30}H_{36}O_3$: 444.6051).

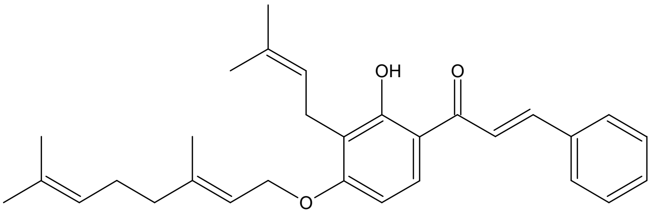

28

(2*E*)-1-[4-(farnesyloxy)-2-hydroxy-3-(prenyl)phenyl]-3-phenylprop-2-en-1-one (**29**): The compound was isolated as a yellow solid in a yield of 69.6% and a melting point of 62-64 °C. ^{1}H NMR (400 MHz, $CDCl_3$): δ 13.37 (*s*, 1H, 2′-OH), 7.87 (*d*, *J* =15.5 Hz, 1H, H-6′); 7.77 (*d*, *J* =9.1 Hz, 1H, H-8); 7.63 (*m*, 2H, H-7 and H-4); 7.42 (*m*, 4H, H-2, H-3, H-5 and H-6); 6.49 (*d*, *J* =9.0 Hz, 1H, H-5′); 5.48 (*m*, 1H, H-2′′); 5.25 (*m*, 1H, H-8′); 5.08 (*m*, 2H, H-6′′ and H-10′′); 4.65 (*d*, *J* =6.6 Hz, 2H, H-1′′); 3.39 (*d*, *J* =7.2 Hz, 1H, H-7′); 2.09 (*m*, 4H, H-4′′, H-5′′, H-8′′ and H-9′′); 1.80 (*s*, 6H, H-10′ and H-13′′); 1.75 (*s*, 3H, H-11′); 1.67 (*s*, 6H, H-14′′ and H-15′′). ^{13}C NMR (100 MHz, $CDCl_3$): δ 192.1 (C-9); 163.2 (C-2′); 162.9 (C-4′); 144.0 (C-7); 141.3 (C- 3′′);135.3 (C-7′′); 134.9 (C-1); 131.5 (C-9′ and C-11′′); 131.3 (C-6′); 130.5 (C-4); 129.0 (C-2 and C-6); 128.5 (C-3 and C-5); 124.3 (C-6′′); 123.6 (C-10′′); 122.1 (C-8); 120.7 (C-8); 119.3 (C-2′′) 117.9 (C-3′); 114.5 (C-1′); 103.3 (C-5′); 65.4 (C-1′′); 39.7 (C-4′′); 39.5 (C-8′′); 26.7 (C-9′′); 26.2 (C-5′′); 25.8 (C-12′′); 25.7 (C-11′); 21.8 (C-7′); 17.9 (C-10′); 17.7 (C-15′′); 16.7 (C-14′′); 16.0 (C-13′). HRMS: $[M + H]^+$ ion m/z 513.7351 ($C_{35}H_{44}O_3$: 512.7220).

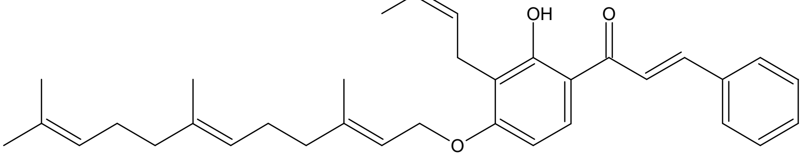

29

S3. NMR ^{1}H and ^{13}C of compounds 30-35.

^{1}H NMR (400 MHz, $CDCl_3$) spectrum of compound 30

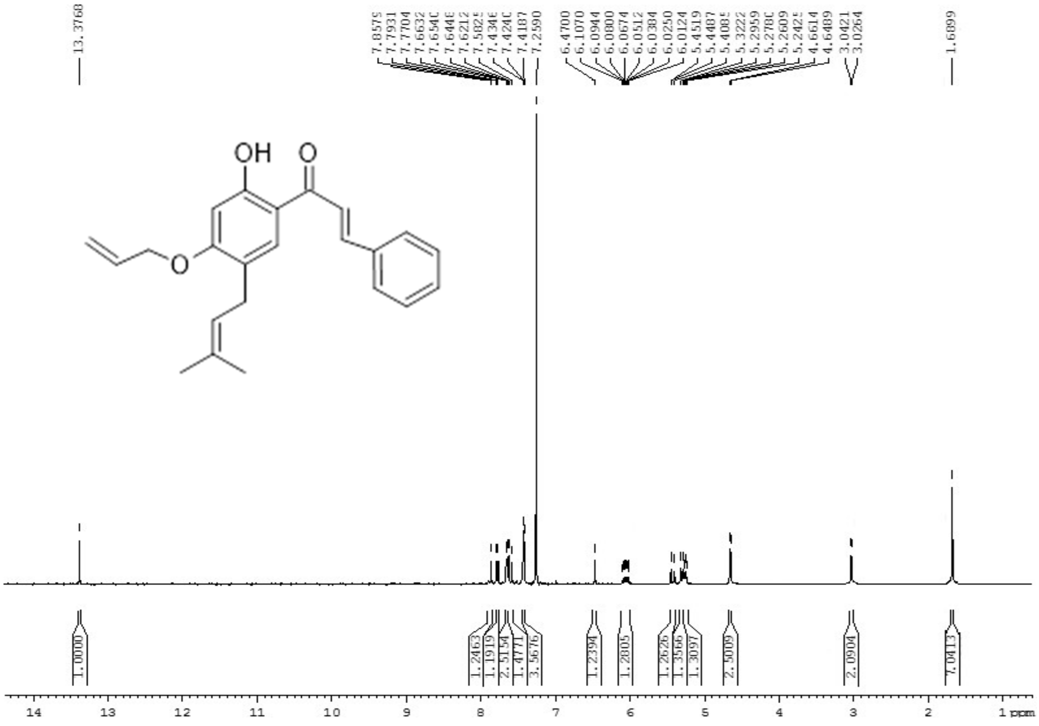

^{13}C NMR (100 MHz, $CDCl_3$) spectrum of compound 30

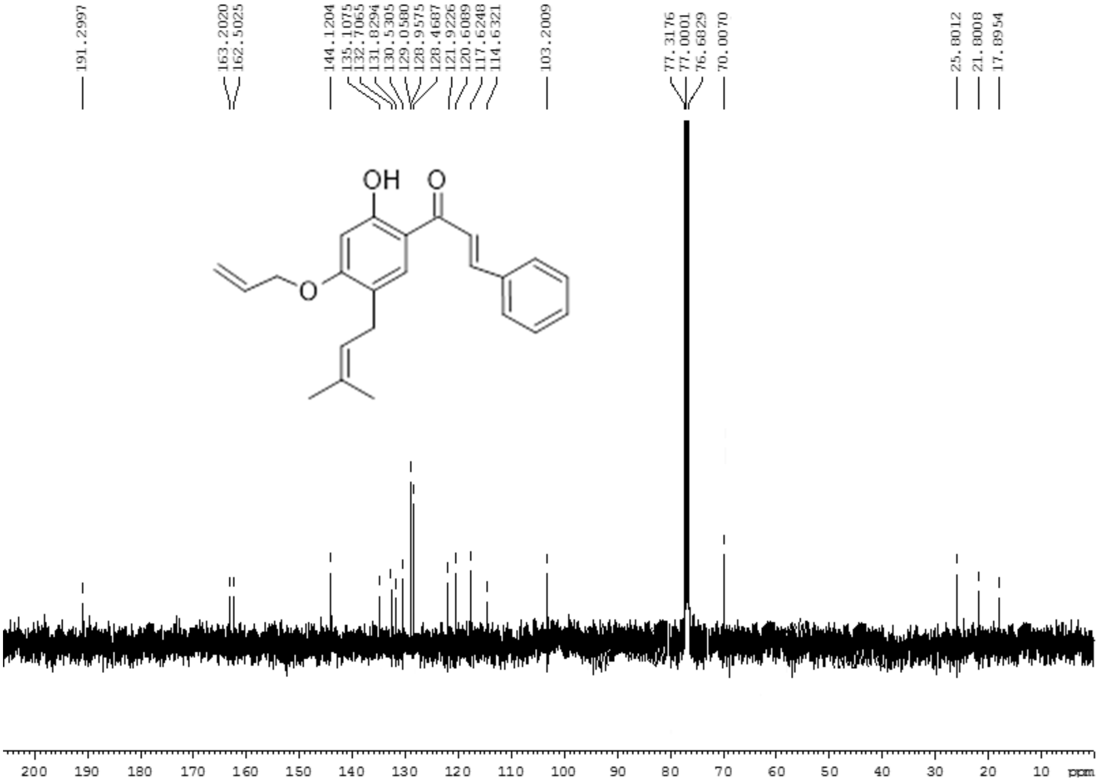

^{1}H NMR (400 MHz, CDCl₃) spectrum of compound 31

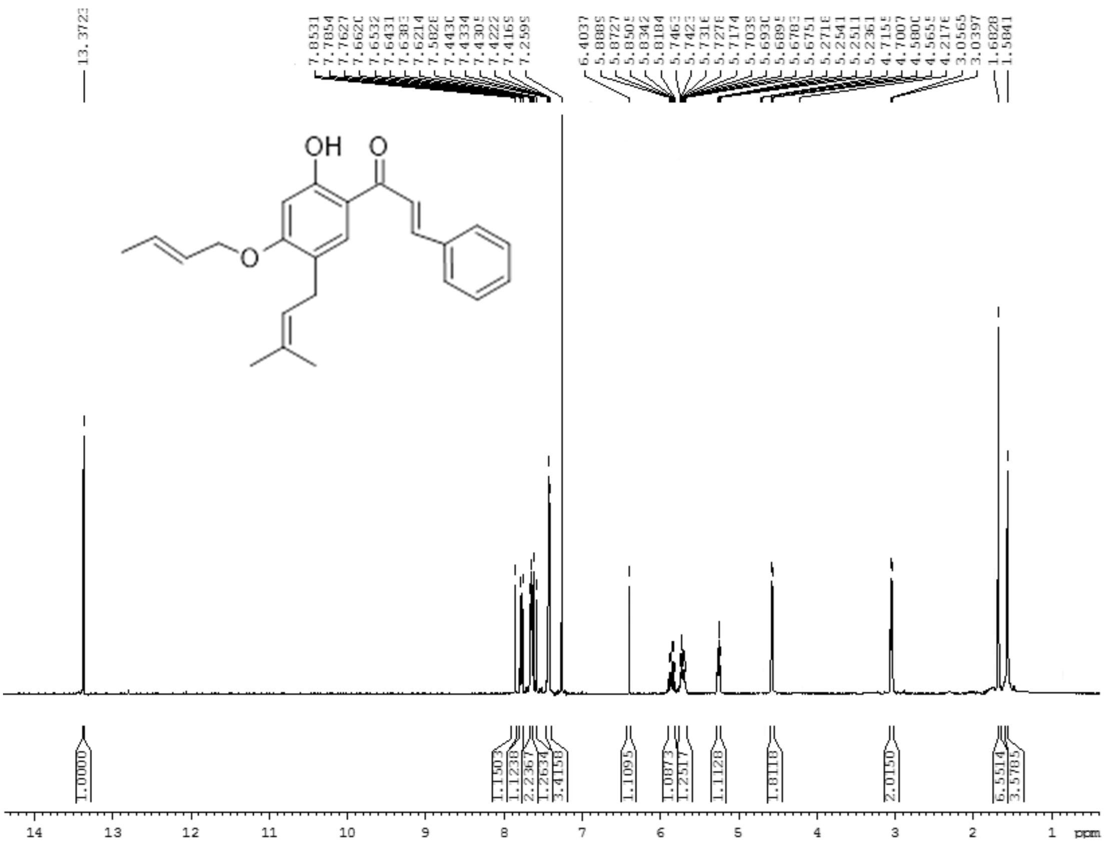

^{13}C NMR (100 MHz, $CDCl_3$) spectrum of compound 31

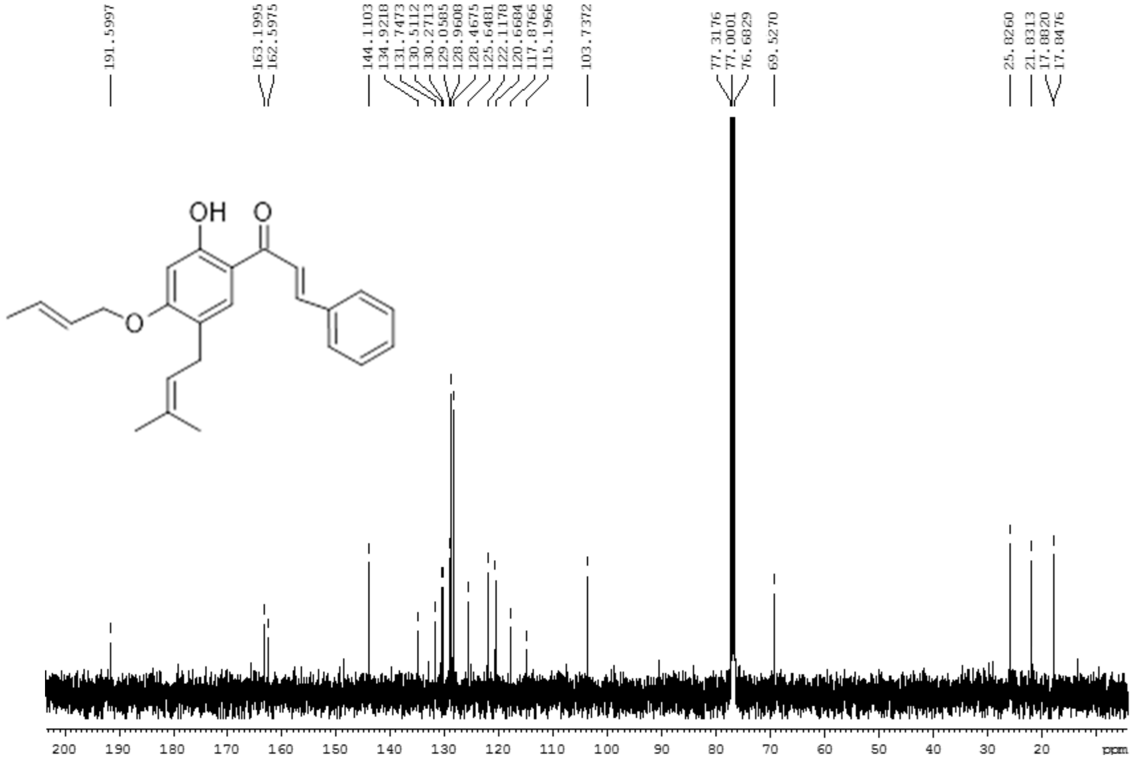

^{1}H NMR (400 MHz, CDCl₃) spectrum of compound 32

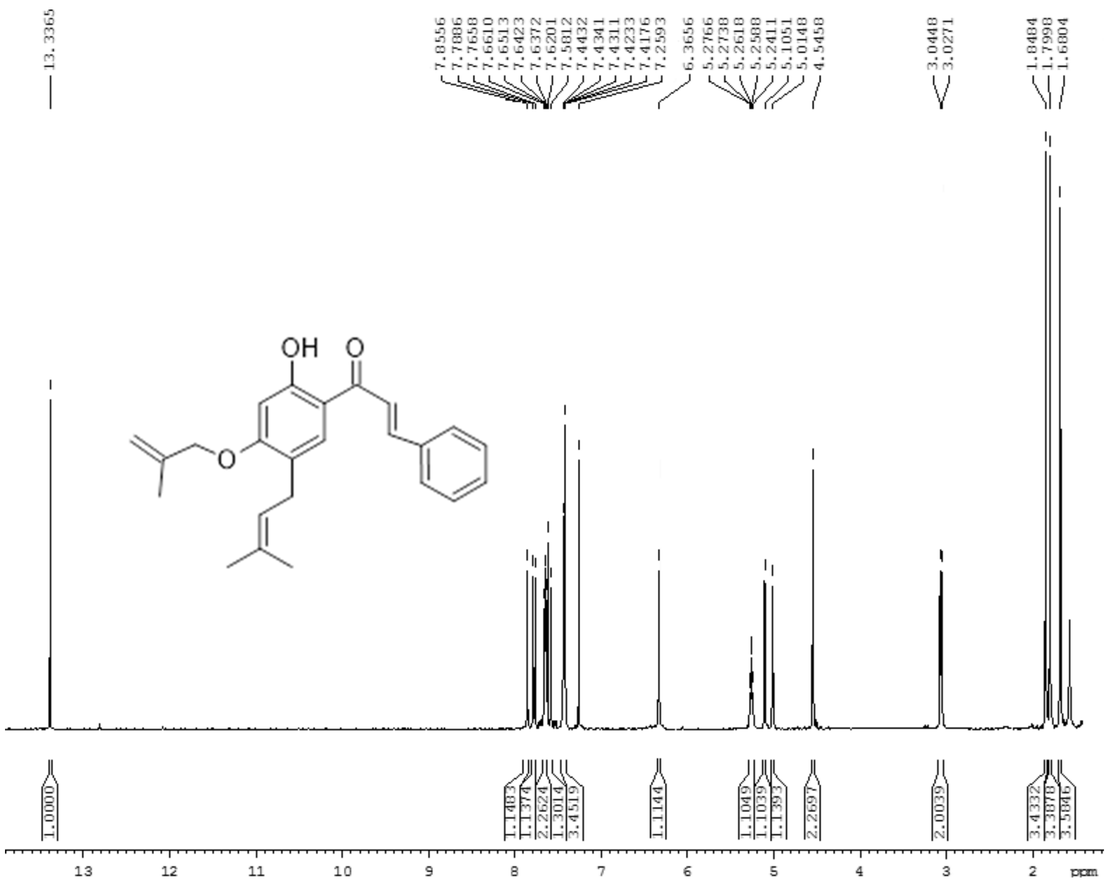

^{13}C NMR (100 MHz, $CDCl_3$) spectrum of compound 32

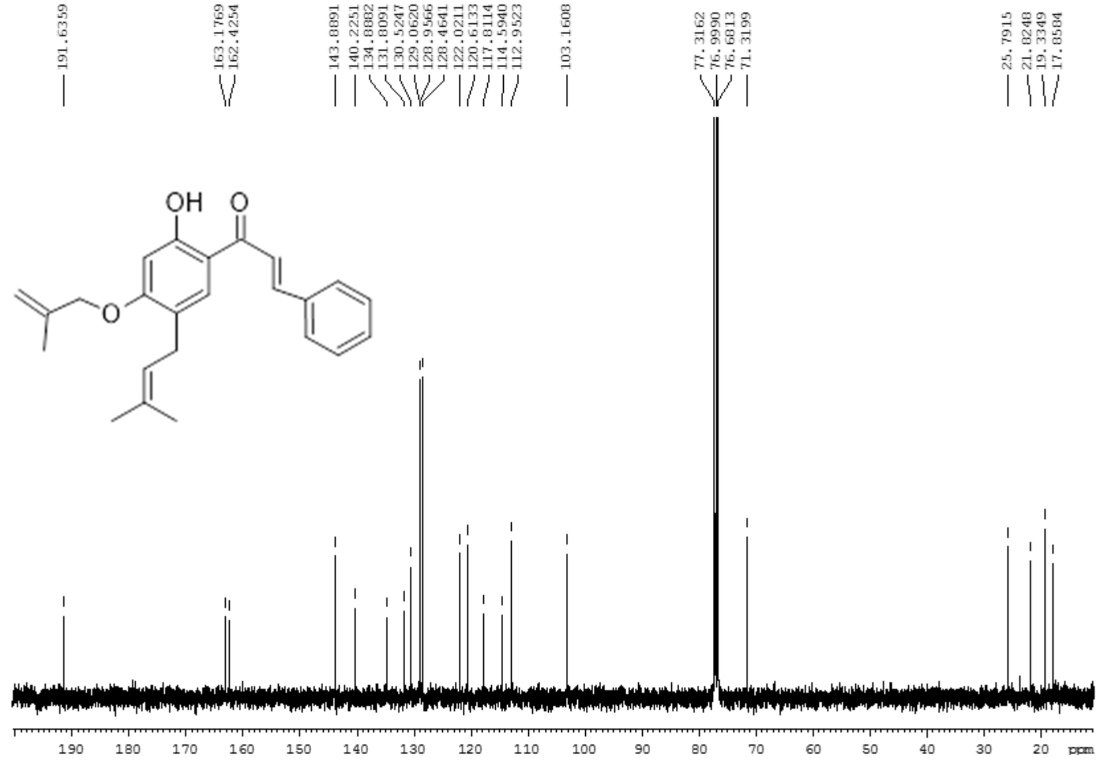

^{1}H NMR (400 MHz, $CDCl_3$) spectrum of compound 33

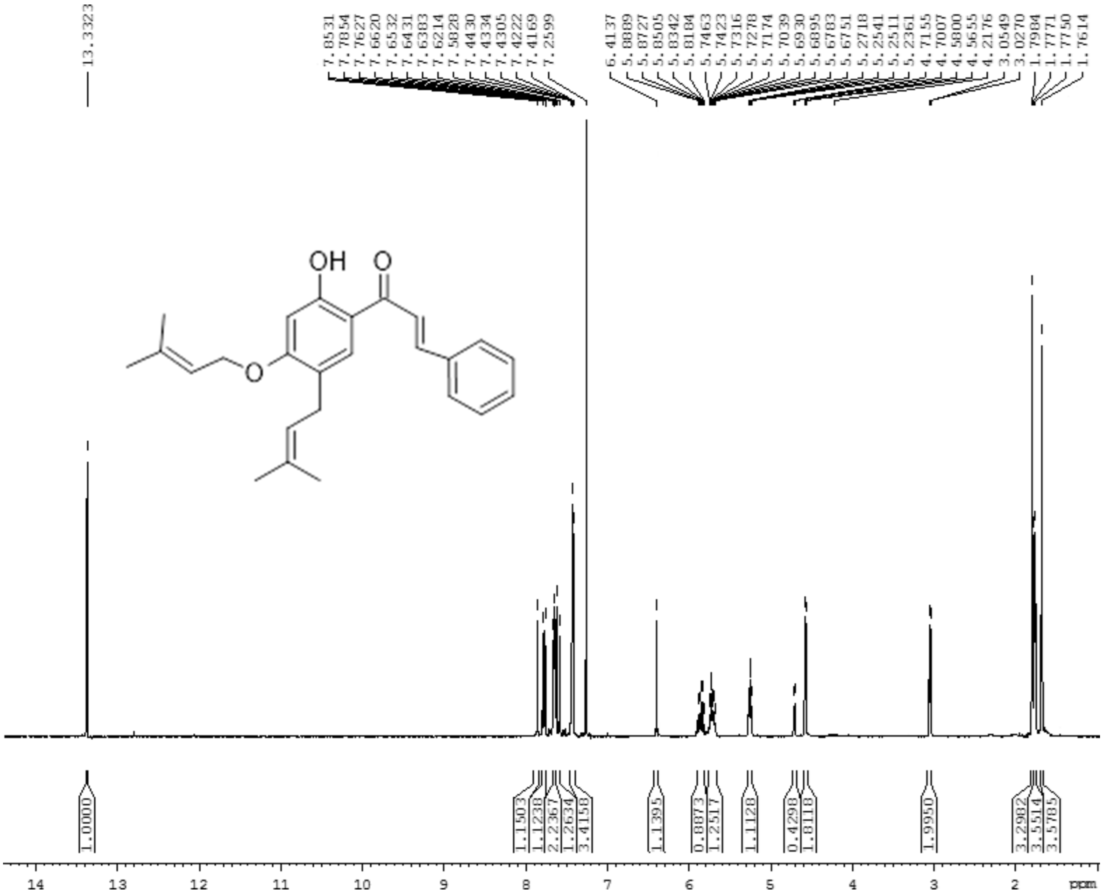

^{13}C NMR (100 MHz, CDCl$_3$) spectrum of compound 33

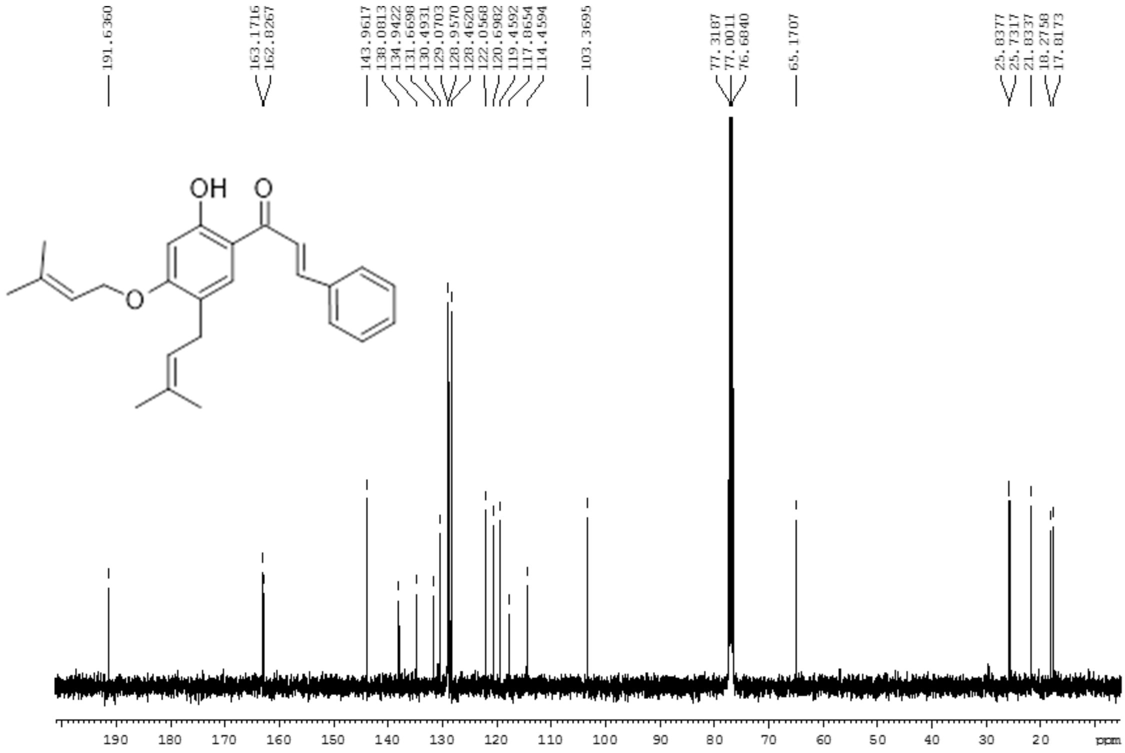

^{1}H NMR (400 MHz, $CDCl_3$) spectrum of compound 34

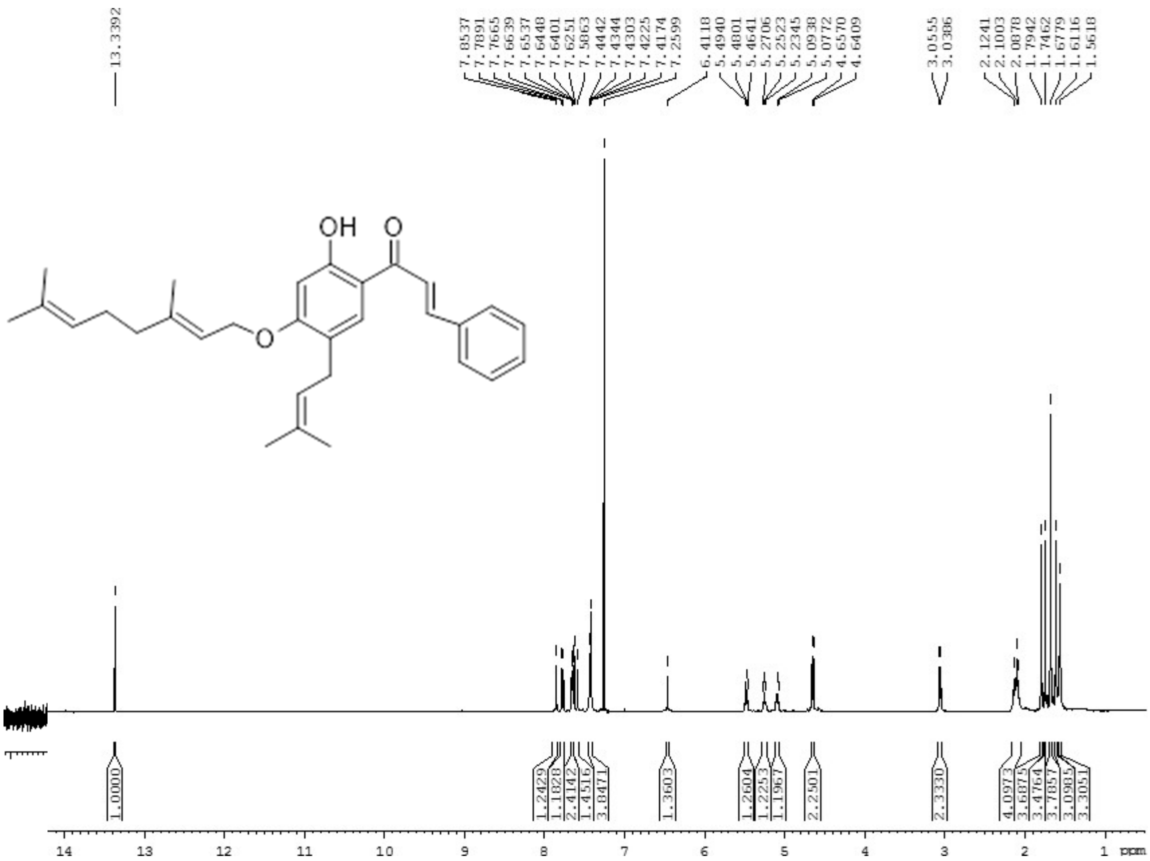

^{13}C NMR (100 MHz, $CDCl_3$) spectrum of compound 34

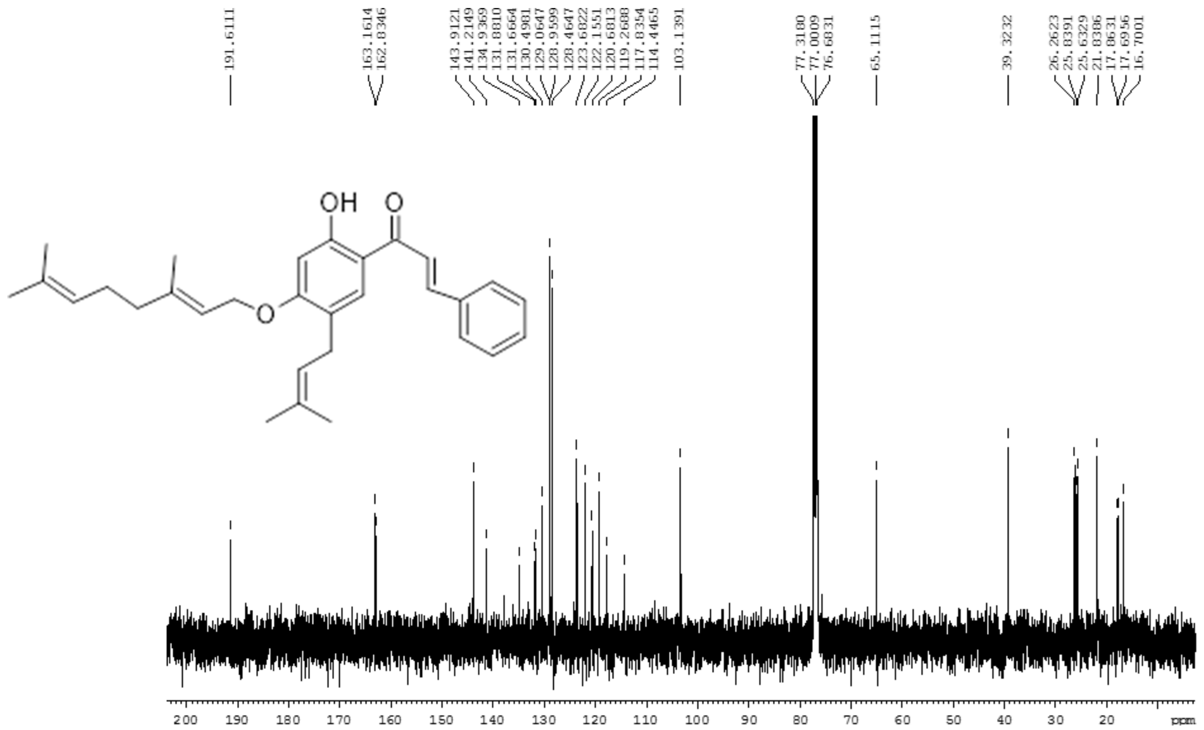

^{1}H NMR (400 MHz, CDCl₃) spectrum of compound 35

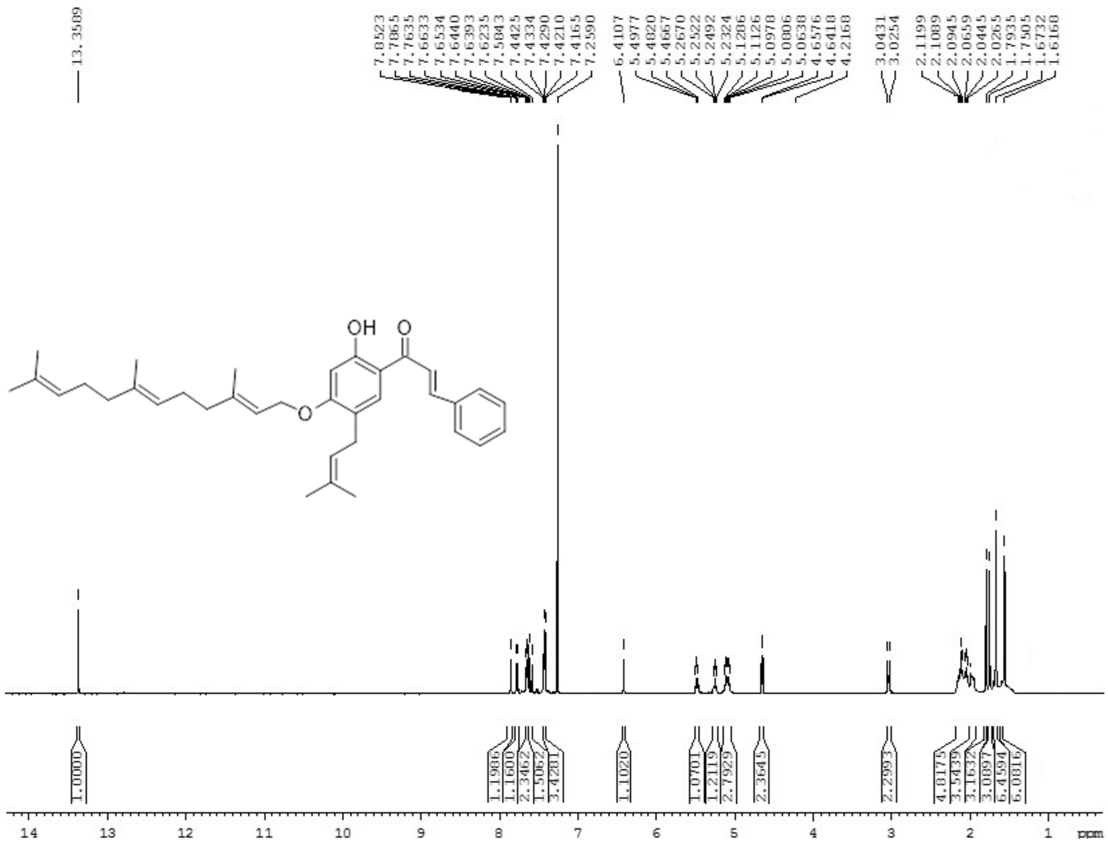

^{13}C NMR (100 MHz, CDCl$_3$) spectrum of compound 35

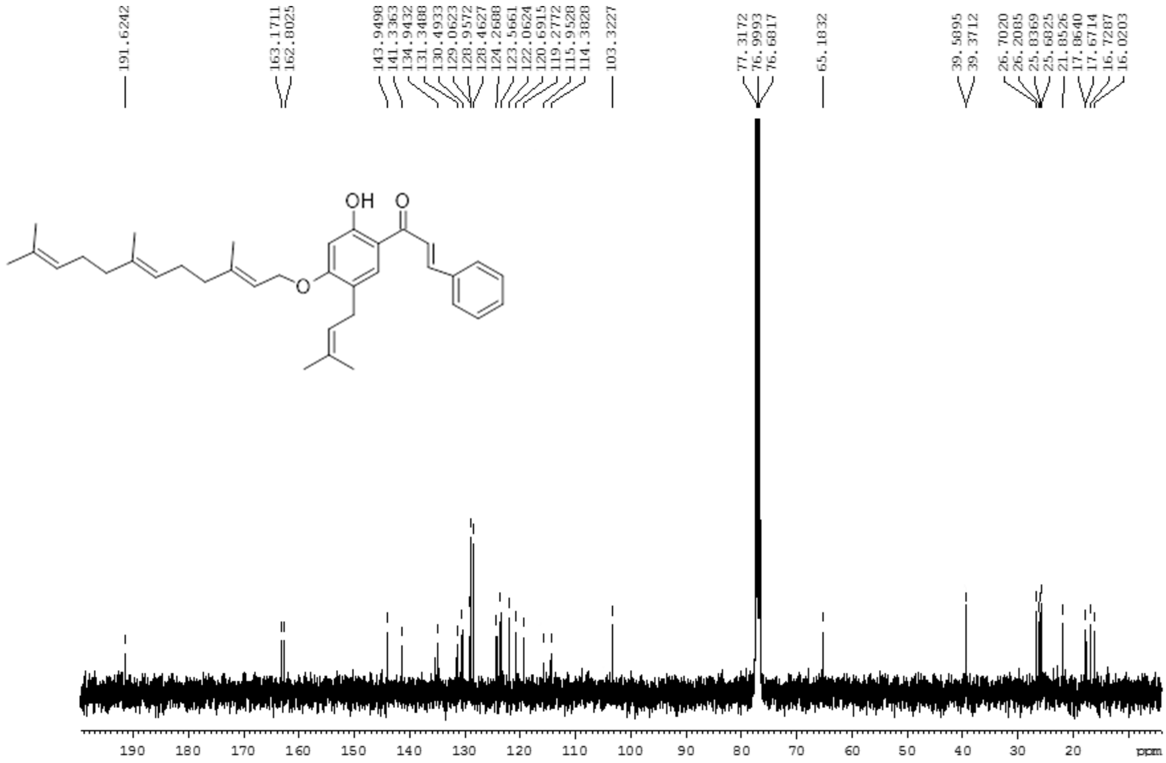
191.6242
163.1711
162.8025
143.9498
141.3363
134.9432
131.3488
130.4933
129.0623
128.9572
128.4627
124.2688
123.5661
122.0624
120.6915
119.2772
115.9528
114.3828
103.3227
77.3172
76.9993
76.6817
65.1832
39.5895
39.3712
26.7020
26.2085
25.8369
25.6825
21.8526
17.8640
17.6714
16.7287
16.0203
OH
O
O
190 180 170 160 150 140 130 120 110 100 90 80 70 60 50 40 30 20 ppm
